# Integrative analysis of loss-of-function variants in clinical and genomic data reveals novel genes associated with cardiovascular traits

**DOI:** 10.1186/s12920-019-0542-3

**Published:** 2019-07-25

**Authors:** Benjamin S. Glicksberg, Letizia Amadori, Nicholas K. Akers, Katyayani Sukhavasi, Oscar Franzén, Li Li, Gillian M. Belbin, Kristin L. Akers, Khader Shameer, Marcus A. Badgeley, Kipp W. Johnson, Ben Readhead, Bruce J. Darrow, Eimear E. Kenny, Christer Betsholtz, Raili Ermel, Josefin Skogsberg, Arno Ruusalepp, Eric E. Schadt, Joel T. Dudley, Hongxia Ren, Jason C. Kovacic, Chiara Giannarelli, Shuyu D. Li, Johan L. M. Björkegren, Rong Chen

**Affiliations:** 1 Department of Genetics and Genomic Sciences, The Icahn Institute for Genomics and Multiscale Biology, Icahn School of Medicine at Mount Sinai, One Gustave L. Levy Place, New York, NY 10029 USA; 2 The Institute for Next Generation Healthcare, Icahn School of Medicine at Mount Sinai, One Gustave L. Levy Place, New York, NY 10029 USA; 3 Cardiovascular Research Center and Cardiovascular Institute, Icahn School of Medicine at Mount Sinai, One Gustave L. Levy Place, New York, NY 10029 USA; 4 Department of Pathophysiology, Institute of Biomedicine and Translation Medicine, University of Tartu, Biomeedikum, Ravila 19, 50411 Tartu, Estonia; 5 Clinical Gene Networks AB, Jungfrugatan 10, 114 44 Stockholm, Sweden; 6 Charles Bronfman Institute of Personalized Medicine, Icahn School of Medicine at Mount Sinai, One Gustave L. Levy Place, New York, NY 10029 USA; 7 Sema4, a Mount Sinai venture, Stamford, CT 06902 USA; 8 Department of Preventive Medicine, Icahn School of Medicine at Mount Sinai, One Gustave L. Levy Place, New York, NY 10029 USA; 9 Department of Immunology, Genetics and Pathology, Uppsala University, 751 85 Uppsala, Sweden; 10 Department of Cardiac Surgery, Tartu University Hospital, 1a Ludwig Puusepa Street, 50406 Tartu, Estonia; 11 Integrated Cardio Metabolic Centre, Department of Medicine, Karolinska Institutet, Karolinska Universitetssjukhuset Huddinge, 141 86 Stockholm, Sweden; 12 Department of Health Policy and Research, Icahn School of Medicine at Mount Sinai, One Gustave L. Levy Place, New York, NY 10029 USA; 13 Department of Pediatrics, Herman B Wells Center for Pediatric Research, Center for Diabetes and Metabolic Diseases, Stark Neurosciences Research Institute, Indiana University, 635 Barnhill Dr., MS2049, Indianapolis, IN 46202 USA; 14 Bakar Computational Health Sciences Institute, University of California San Francisco, San Francisco, 94158 CA USA; 15 Integrated Cardio Metabolic Centre, Department of Medicine, Karolinska Institutet, Novum, 14157 Huddinge, Sweden

**Keywords:** Loss-of-function variant, Cardiovascular traits, Genetic association, Integrative data analysis, Target identification and validation, Electronic Medical Records

## Abstract

**Background:**

Genetic loss-of-function variants (LoFs) associated with disease traits are increasingly recognized as critical evidence for the selection of therapeutic targets. We integrated the analysis of genetic and clinical data from 10,511 individuals in the Mount Sinai Bio*Me* Biobank to identify genes with loss-of-function variants (LoFs) significantly associated with cardiovascular disease (CVD) traits, and used RNA-sequence data of seven metabolic and vascular tissues isolated from 600 CVD patients in the Stockholm-Tartu Atherosclerosis Reverse Network Engineering Task (STARNET) study for validation. We also carried out in vitro functional studies of several candidate genes, and in vivo studies of one gene.

**Results:**

We identified LoFs in 433 genes significantly associated with at least one of 10 major CVD traits. Next, we used RNA-sequence data from the STARNET study to validate 115 of the 433 LoF harboring-genes in that their expression levels were concordantly associated with corresponding CVD traits. Together with the documented hepatic lipid-lowering gene, *APOC3,* the expression levels of six additional liver LoF-genes were positively associated with levels of plasma lipids in STARNET. Candidate LoF-genes were subjected to gene silencing in HepG2 cells with marked overall effects on cellular LDLR, levels of triglycerides and on secreted APOB100 and PCSK9. In addition, we identified novel LoFs in *DGAT2* associated with lower plasma cholesterol and glucose levels in Bio*Me* that were also confirmed in STARNET, and showed a selective DGAT2-inhibitor in C57BL/6 mice not only significantly lowered fasting glucose levels but also affected body weight.

**Conclusion:**

In sum, by integrating genetic and electronic medical record data, and leveraging one of the world’s largest human RNA-sequence datasets (STARNET), we identified known and novel CVD-trait related genes that may serve as targets for CVD therapeutics and as such merit further investigation.

**Electronic supplementary material:**

The online version of this article (10.1186/s12920-019-0542-3) contains supplementary material, which is available to authorized users.

## Background

The delineation and association of loss-of-function variants (LoFs) with human diseases and phenotypic traits continues to play an increasingly important role in the discovery and validation of novel therapeutic targets. Nonsense mutations in *PCSK9* are associated with an 88% reduction in the risk of coronary heart disease (CHD) [1]. While large clinical trials are ongoing [2], current evidence suggests that PCSK9 inhibitors not only lower LDL cholesterol, but also reduce cardiovascular events [3, 4]. In fact, in the recent published FOURIER trial, the PCSK9 inhibitor Evolocumab used in conjunction with background of statin therapy was shown to significantly reduce the risk of cardiovascular events as well as levels of plasma LDL cholesterol [5]. In another example, LoF mutations in *NPC1L1,* encoding a transporter involved in the absorption of dietary cholesterol, are associated with reduced incidence of CHD [6], and a small-molecule inhibitor of NPC1L1, ezetimibe, was found to both lower plasma LDL levels and reduce the risk of CHD events [7]. *HMGCR*, *LDLR*, and *APOC3* are additional examples of genes with LoFs or other genetic variants where carriers show lower levels of plasma LDL or triglycerides and a reduced incidence of CHD [8, 9]. It is also well known that statin therapy targeting HMGCR reduces risk of both primary and recurrent CHD events [10]. Hence, the evidence that human genetics may improve therapeutic target selection is mounting and increasingly recognized. In fact, retrospective analysis shows that for novel targets with human genetic validation, the rate of success in clinical development is increased twofold [11].

Besides the targeted analysis of human LoFs in candidate genes, several systematic surveys of LoFs [12, 13] and their associations with clinical phenotypes [14–18] have been performed including the recent DiscovEHR study [19] where the distribution and clinical impact of LoFs in 50,726 whole exomes were investigated. A common theme for these studies is that LoF-phenotype associations were found both in established disease-trait genes, such as *PCSK9* (with plasma LDL levels) and *APOC3* (with plasma triglycerides)*,* as well as in novel genes associated with unexpected clinical traits [15, 19]. Thus, systematically discovering LoF-harboring genes associated with clinical traits appears to be an effective approach towards precision medicine [citation: https://academic.oup.com/hmg/article/27/R1/R56/4969371] by identifying novel disease candidate genes that may prove useful as drug targets.

**Fig. 1:**
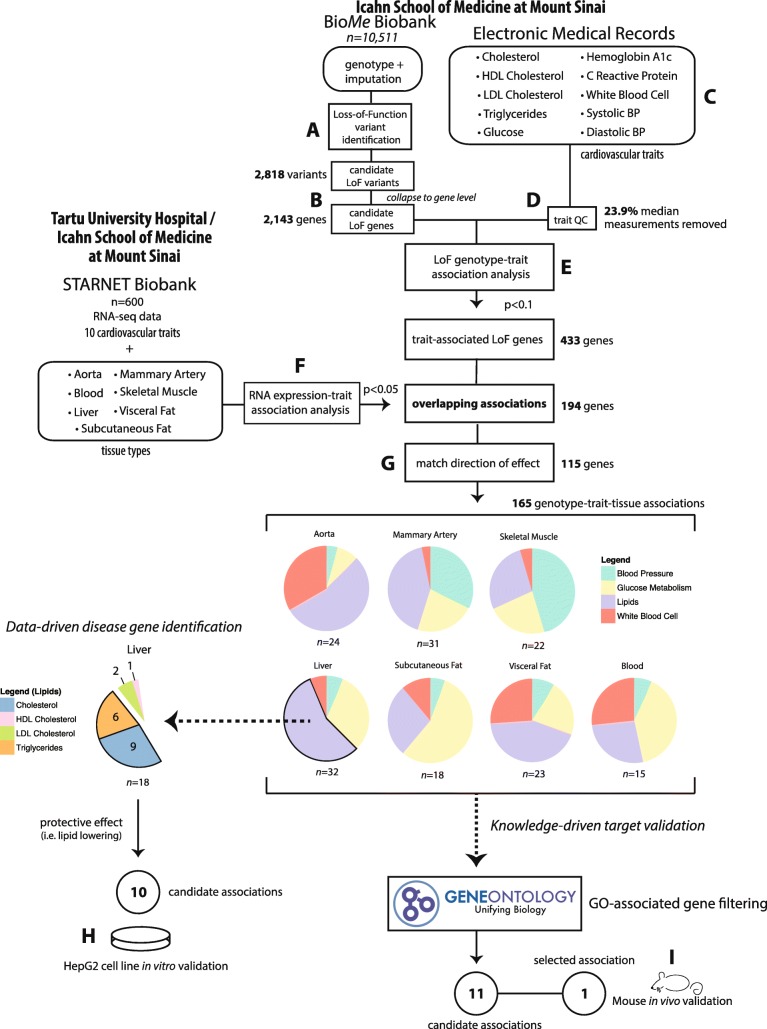
Overall workflow of the study. We first identified high-confidence LoFs in genotyping and the imputed data from the Bio*Me* Biobank (**a**). Then for each gene with a LoF, we partitioned the Bio*Me* individuals into LoF carriers and non-carriers (**b**) for comparison of 10 CVD-related traits obtained from the Mount Sinai Hospital Electronic Medical Records (MSH-EMR) (**c**). Next, we performed trait-specific quality-control (QC) by considering that the CVD-traits are affected by certain ongoing medications (**d**), followed by statistical analyses to robustly identify LoF-genes that were significantly associated with at least one of these CVD traits (**e**). In the next step (**f**), we assessed LoF-harboring genes associated with any of the CVD traits by exploring associations between RNA expression levels of these genes and corresponding CVD traits across seven tissues in STARNET [27]. We then selected genes with concordant CVD trait-associations in both Bio*Me* and STARNET (i.e., when LoFs in a gene are associated with low values of a CVD trait, low expression of the same gene is also associated with low values of the trait) (**g**). For LoF genes associated with lower plasma cholesterol or triglyceride levels in Bio*Me* Biobank and STARNET liver data, we carried out functional in vitro evaluation using HepG2 cells (H). Last, a knowledge-driven filtration approach was used for leveraging information in Gene Ontology (GO) to select potential therapeutic targets for validation in mice (**i**)

In the current study, we identified LoF variants with possible implications for cardiovascular disease (CVD) using Mount Sinai Bio*Me* Biobank, established in 2007 in New York City, an ongoing, broadly-consented Electronic Medical Record (EMR)-linked data repository that enrolls patients non-selectively from the Mount Sinai Medical Center. So far, over 34,000 ancestrally diverse participants have been enrolled, of which a subset of 10,511 with genotype data were used here. Subjects have been extensively characterized with longitudinal clinical information in EMRs, including disease diagnoses, laboratory test results, and medication history [20, 21]. We have demonstrated successful utility of these data for disease subtyping [22], automated phenotyping [citation: https://www.worldscientific.com/doi/abs/10.1142/9789813235533_0014], comorbidity analyses [23, 24], health assessment via real-time visualization [25], and drug repurposing [26]. To validate CVD-trait gene associations detected in Bio*Me*, we used RNA sequencing (RNAseq) data from blood and six vascular and metabolic tissues in 600 well-characterized coronary artery disease (CAD) patients of the Stockholm-Tartu Atherosclerosis Reverse Network Engineering Task (STARNET) study [27]. A schematic flow of how we identified LoFs in the BioMe Biobank, further evaluated corresponding LoF-genes in STARNET and then undertook in vitro and in vivo functional validation is shown in Fig. 1.

## Methods

### Bio*Me* research cohort

In the current study, we utilized a genotyped biobank cohort within the Icahn School of Medicine at Mount Sinai. The Charles Bronfman Institute for Personalized Medicine (http://icahn.mssm.edu/research/institutes/ipm) Bio*Me* Biobank is a repository that consists of over 34,000 enrolled participants. Currently, 11,212 individuals are genotyped using the Illumina Human Omni Express Exome Bead-8 BeadChip v1.1, which has 866,864 markers per sample. Bio*Me* actively recruits individuals from upper Manhattan facilitating strong representation of ethnic minorities often underrepresented in other studies. Out of the genotyped individuals, there were 6857 (61.2%) women and 4350 (38.8%) men, with three individuals removed due to unknown sex. The self-reported ancestry breakdown is as follows: 2090 (18.6%) European, 3763 African American (33.6%), 5194 Hispanic/Latino (46.3%), and 134 Other (0.01%). Due to low representation, we merged the remaining ancestry groups into the “Other” category. The mean age for the Bio*Me* participants was 55.8 ± 15.8 years.

### Genotype imputation, quality control, and filtration

For the genotype data, all individuals had call rates above 99% and all SNPs had call rates > 95%, at least one minor allele, and Hardy–Weinberg equilibrium *p*-value >5e-05. We performed phasing on the genotype data using SHAPEIT v2 (r644) [28] and imputation using IMPUTE2 v2.3 [29] (chunk size of 5 Mb). For the imputation reference panel, we used the 1000 Genomes [30] Phase 1 version 3 variant set (August, 2012; https://mathgen.stats.ox.ac.uk/impute/data_download_1000G_phase1_integrated.html). We then performed several quality control steps on the over 37 million imputed SNP data: 1. We removed any sites with < 0.3 gen INFO score (from IMPUTE2); 2. We discarded any variants with < 90% site completeness; 3. We removed individual allele call below 95% genotype probability.

We used PLINK [31] to identify related individuals (relatives) in the cohort that could bias the results of subsequent analyses. We identified 612 pairs of individuals (1224 total) that had PI-HAT scores > 0.2, which indicates direct relatedness. Accordingly, we randomly selected an individual from each pair to remove from our cohort. After all of the QC steps detailed, the cohort contained 10,511 individuals.

### Variant annotation procedure

As different annotators have their strengths and weaknesses, we decided to utilize multiple annotators to identify predicted LoF mutations. All annotators were run using the hg19 build. Variant Annotation Tool (VAT) is an annotator that was used in the seminal paper by MacArthur et al. [12] to identify LoF mutations. We obtained the VAT annotated file from 1000 genomes [30] ftp server (ftp://ftp-trace.ncbi.nih.gov/1000genomes/ftp/phase1/analysis_results/functional_annotation/annotated_vcfs/), specifically phase 1, version 3, where full documentation can be found. We limited the variants to those that putatively cause LoF by restricting “effect_type” to “frameshift_indel”, “stop_gained”, or “splice_site”. We also restricted these variants to those with predicted “full” effect. These filtering steps resulted in 4757 VAT-predicted LoF mutations for our dataset.

ANNOVAR is another variant annotator tool utilized in a related follow-up study by Li et al. [15]. Like with the previous annotator, we ran our dataset mimicking the same parameters used in their study to identify LoFs using ANNOVAR (v. 2015Apr14). Specifically, we filtered the resulting “Gene-based Annotation” results for variants causing “frameshift substitution”, “stopgain”, or “exonic;splicing”. We obtained 10,582 predicted LoFs from these filtering steps.

SnpEff [32] is a popular annotation tool that has been used in many studies. To identify LoF mutations, we ran our dataset using this tool (SnpEff v. 3.6) and filtered for SNPs with “HIGH” Effect_Impact, “protein-coding” Transcript_BioType, and the following Effect types: “STOP_GAINED”, “SPLICE_SITE_ACCEPTOR”, “SPLICE_SITE_DONOR”, or “FRAME_SHIFT”. We identified 6387 putative LoFs from our dataset using these criteria.

### Consensus LoF variant classification

We only included into our analyses consensus mutations, or mutations in which at least two of three annotators identify as LoF. Additional file 1: Figure S1 demonstrates the relative distribution of LoF annotation between the three annotators. It is important to note that the overall numbers of variants called per annotator might differ from the effect calls per annotator as a particular variant can have more than one effect call. In total, we identified 6421 putative consensus LoF variants. We next performed extensive, further QC curation to ensure high confidence and quality LoF annotation. From the imputation-specific QC, we only kept variants that had > 0.3 gen INFO scores (IMPUTE2; 1666 failed) and > 90% site completeness using 95% as call threshold (301 failed). Furthermore, we excluded low confidence alleles with quality scores < 95%. Additionally, we only included variants that: 1) had consensus LoF annotation on at least one overlapping transcript (i.e., only include variant if two or more annotators classified the same associated transcript as LoF; 28 failed); 2) were not in the final exon of the predicted LoF transcript (1498 failed); 3) had < 2% alternate allele frequency (362 failed). Taken together, these filtering steps resulted in the removal of 2029 unique variants (as a single variant could fail overlapping filtering steps), leaving 3392 unique remaining variants from the original list of 6421. Lastly, we filtered variants in which there were no carriers in our cohort, which produced 2818 high confidence predicted LoF variants for use in our study (see Additional file 1: Figure S2A for variant filtering procedure overview).

**Fig. 2:**
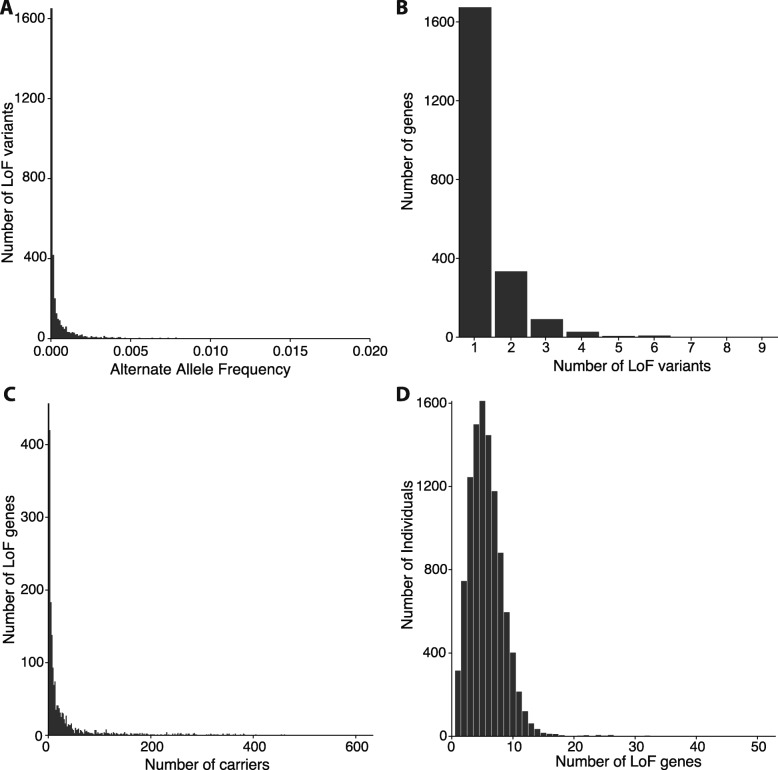
The predicted LoF variants in the Bio*Me* Biobank cohort. **a** Allele frequency of all LoF variants in Bio*Me* Biobank population. **b** Distribution of the number of LoF variants per gene. **c** Carrier frequency of LoF genes. **d** Number of LoF genes carried per person

As a matter of practicality, we collapsed mutations to the gene level, as the predicted outcome of each independent LoF mutation within a single gene is theoretically identical. The relative distributions of mutations per gene can be seen in Fig. 2b. On average, we found 1.32 ± 0.73 (STD) LoF mutations per gene, with a maximum of 9 mutations in the gene *PZP*, which has 65,552 base pairs. The frequency of these mutations in the population can be seen in Fig. 2c. Specifically, the 2818 variants were collapsed into a useable list of 2143 genes. The distribution of LoF gene frequency in this population can be seen in Fig. 2d. We present all variants used in this study along with their frequency in our cohort and their respective effect call for each annotator in Additional file 2: Table S1.

### Trait data collection and processing

The Bio*Me* Biobank cohort used in this study is connected to the MSH’s EMR via de-identified codes managed by the Mount Sinai Data Warehouse. These records contain information on trait measurements and medication prescriptions, which we collected for all of the individuals in the cohort. We selected 10 CVD-related traits for analysis, specifically: glucose levels, hemoglobin A1c, total cholesterol, LDL cholesterol, HDL cholesterol, triglycerides, high sensitivity C reactive protein, diastolic blood pressure, systolic blood pressure, and white blood cell count. We obtained trait measurements through querying the EMR, which consisted of individual, value, date, and unit of measurement. This cohort, however, was not specifically screened for enrollment criteria. As such, there are many factors that could have extreme effects on the trait values that are irrespective of genetics. Of crucial concern are changes in trait levels due to diseases and/or medication use. While several algorithms exist to control the effect of medications in trait analytics, there is no established, universal methodology. Accordingly we devised a thorough and individualized procedure to control for confounding factors, including disease and medication effects, in the current study, which we describe in detail in the Additional file 1: Supplementary Materials.

In brief, we performed several QC steps on the trait measurement data to ensure the highest achievable validity (Additional file 1: Figure S2B). We filtered out all incorrectly labeled, outlier, and medication/disease-affected measurements, and then combined any remaining measurements into a single median value per person. For each of the 10 traits, we present the exclusion QC process (i.e. number of individuals and total number of measurements before and after filtering) in Additional file 2: Tables S3-S5; specifically due to medication use (Additional file 2: Table S3), outliers (Additional file 2: Table S4), and combined (Additional file 2: Table S5).

### Association analysis between LoF genes and traits

We performed linear regression analysis to test whether having a LoF mutation significantly affects trait measurements. Our analytical goal was to assess if genes harboring LoF genetic variants were significantly associated with CVD traits. Therefore, we chose to perform the analysis at gene level rather than at the level of individual variants. CVD trait values and ages used in the analyses were median values that passed QC steps explained previously. We observed the regression residuals were not approximately normally distributed and thus would not satisfy assumptions of linear regression. Accordingly, we first performed the Box-Cox transformation (Eq. 1) procedure to identify the optimal transformation parameter per trait that produces a normal distribution of residuals by sequencing through λ values from the range [− 2, 2] in increments of 0.5. The natural logarithm was used for the case of λ = 0. For each trait, we selected the λ value corresponding to the highest regression model (Eq. 2 without the gene variable) log-likelihood as the optimal transformation parameter. All regressions were generalized linear models fit to the data using the method of iteratively reweighted least squares. The effects of transformation can be found in Additional file 1: Figure S8.

1 $$ {y}_i^{\left(\lambda \right)}\left\{\begin{array}{c}\frac{y_i^{\left(\lambda \right)}-1}{\lambda}\kern1em if\lambda \ne 0\\ {}\ln \left({y}_i\right)\kern1em if\lambda \ne 0\end{array}\right. $$

With the lambda parameter calculated, we then performed the following formula to assess for genotype-trait significance:

2 $$ P\left({\mathrm{trait}}^{\lambda }|{\beta}_0+{\beta}_{\mathrm{l}}\cdot \mathrm{gene}+{\beta}_{\mathrm{g}}\cdot \mathrm{sex}+{\beta}_{\mathrm{a}}\cdot \mathrm{age}+{\beta}_{\mathrm{pc}1}\cdot \mathrm{PC}1\dots +\dots {\beta}_{\mathrm{pc}5}\cdot \mathrm{PC}5\right) $$

where trait is continuous value, λ is the transformation parameter factor derived through Box-Cox transformation (Eq. 1), gene is binary piecewise Yes/No indicating presence of LoF mutation, sex is binary piecewise Female/Male, age is continuous constant per year, principal components are continuous.

To obtain an estimate of effect size, for associations that were found to be significant in (Eq. 2), we performed a similar statistical analysis albeit without response transformation and used both β_l_ and t-statistic (β_l_ / β standard error).

### RNA-expression data source: STARNET

The details of the STARNET study have been previously published [27], however in brief: patients diagnosed with CAD undergoing open-heart surgery had up to 7 tissues collected, including aortic root (AOR), whole blood, liver (LIV), mammary artery (MAM), subcutaneous fat (SF), skeletal muscle (SKLM), and visceral fat (VAF). RNA was derived from each tissue and sequenced at read lengths of 50 or 100 base pairs from a single-end. Reads were aligned to human genome hg19 using the STAR aligner [33] version 2.3.0e and GENCODE v19 [34] annotations. Reads aligned to genes were summarized using HTSeq [35]. For each tissue, genes with less than one count per million (CPM) in more than 90% of samples were filtered. Genes passing this criteria were normalized using voom [36] using the ‘TMM’ method, generating log (CPM) values and precision weights. A linear model was then used to adjust for batch effects using sequencing flow cell identifier. An lmFit object was generated with the general formula log (CPM) ~ FlowCellID. The residuals of this linear model were used as batch-normalized gene expression values in subsequent analysis.

### Association analysis between gene expression and traits

We compared the RNA expression levels of the identified LoF genes in all STARNET tissues to the same 10 CVD traits. Linear modeling was performed to assess the correlation between gene expression and clinical trait, after adjustment for basic clinical covariates. The model used was: Trait ~ Gene + Age + Gender + BMI.

### Integration of Bio*Me* data and STARNET data

We assumed that LoF variants in Bio*Me* and low gene expression level in STARNET have the same biological and consequently physiological effect. Therefore we defined concordant associations in Bio*Me* and STARNET as follows: when LoFs in a gene are associated with low values of a CVD trait in Bio*Me*, low expression of the same gene is also associated with low values of the trait in STARNET; conversely when LoFs in a gene are associated with high values of a CVD trait in Bio*Me*, low expression of the same gene is also associated with high values of the trait in STARNET. Furthermore, we considered the associations in Bio*Me* and STARNET concordant regardless what tissues in the STARNET data where significant associations between gene expression and CVD-traits were observed. To illustrate the definition of concordant associations in Bio*Me* and STARNET, in Table 2, LoF in gene ACSM3 is associated with low LDL cholesterol level in Bio*Me*, and low gene expression of ACSM3 is also associated with low LDL cholesterol level in skeletal muscle (SKLM) in STARNET; conversely, LoF in gene ACOT11 is associated with high glucose level in Bio*Me*, and low gene expression of ACOT11 is also associated with high glucose level in skeletal muscle (SKLM) in STARNET.

### Mouse *DGAT2* validation procedure

In the current study, C57BL/6 wild type male mice (3 months old, fed chow diet) were obtained from the Jackson Laboratory. The animals were maintained in a pathogen-free and temperature-controlled room on a 12 h light/dark cycle and supplied with food and water ad libitum. Compounds were administered as previously described [37, 38] with modifications, specifically, mice were orally dosed with vehicle (control) or inhibitors 20 mg/kg/day. Body weight and fasting glucose were measured 3 days after compound treatment.

### siRNA candidate targets validation study

Cells from the human hepatocellular carcinoma cell line HepG2 were plated in six-well culture dishes (Corning) containing 10% fetal bovine serum (FBS)-RPMI-1640 medium supplemented with penicillin (100 U/mL) and streptomycin (100 μg/mL). For each gene, cells were transfected with siRNAs (Ambion, Life Technologies, Additional file 2: Table S9), using Lipofectamine RnaiMax according to the manufacturer’s instructions (Invitrogen). Two days after transfection, siRNA-targeted cells and mock-treated controls (scramble siRNA) were incubated with oleic acid (0.75 mM, Sigma Aldrich,) for 24 h in 1% FBS medium. Thereafter, the cells were examined for effects on levels of PCSK9 and LDLR, and on measurements of cholesterol (total cholesterol, TC; and cholesteryl esters, CE) and triglycerides (TG).

Total RNA was isolated from HepG2 cells with the RNeasy Mini-kit (Qiagen) and concentrations determined by NanoDrop (Thermo Scientific). The efficiency of target gene silencing was determined by TaqMan analyses (Additional file 2: Table S9). In brief, cDNA was synthesized from 0.5 μg of total RNA (High-Capacity RNA-to-cDNA Kit, Thermo Scientific) and amplified by real-time PCR with 1xTaqMan universal PCR master mix (Applied Biosystems) using primers and probes from Applied Biosystems (Additional file 2: Table S9); data were normalized to mock control using the comparative Ct method.

#### Cholesterol and triglyceride measurement procedure

Cholesterol accumulation in cell lysates of siRNA-targeted HepG2 cells was measured using the Total Cholesterol and Cholesteryl Ester Colorimetric/Fluorometric Assay kit (BioVision). Lipid levels were normalized by protein concentrations obtained from the same samples using the BCA method (Thermo Scientific). Triglycerides from siRNA targeted HepG2 cells were determined by an enzymatic assay using the Triglyceride Quantification Colorimetric/Fluorometric Kit (BioVision).

#### ELISA assay to measure PCSK9 and LDLR plasma levels

PCSK9 levels were measured using the Human PCSK9 ELISA kit (Cell Biolabs, INC.), according to manufacturer instructions. LDLR levels in cell lysates were measured using the Human LDLR ELISA kit (R&D Systems), according to manufacturer instructions. Absorbance for both methods was quantitated on Promega Glomax reader at 450 nm.

#### Experimental statistical analyses

All experiments were performed in triplicate from 3 independent experiments; data are shown as mean ± SEM. Statistical differences were assessed with unpaired 2-tailed Student t-tests. Values of *P* < 0.05 were considered statistically significant. Computations were performed with GraphPad Prism 6 software (La Jolla, CA 92037 USA).

## Results

### LoF-harboring genes associated with CVD traits in Bio*Me*

Genotyping and imputed data from 11,212 individuals of the Bio*Me* Biobank were used to identify a total of 2818 LoFs in 2143 different genes (Methods; Additional file 1: Figure S1; Additional file 1: Figure S2A). Among the 2818 LoFs, 2117 (75%) were genotyped in the Bio*Me* data, and the rest of the LoFs have been previously identified through direct sequencing in databases such as 1000 Genomes [30] and ExAC [39] (Additional file 2: Table S1). Most of the identified LoFs (95%) were rare (allele frequency < 0.5%, Fig. 2a) and most genes (2009/2143 [94%]) had ≤2 LoFs (Fig. 2b). Approximately 60% of those genes with LoFs had 10 or fewer carriers (Fig. 2c). Among individuals carrying at least one LoF, LoFs were typically found in less than 10 genes (mean 5.7, median 5.0; Fig. 2d).

Next, we selected 10 CVD-traits from the MSH-EMR associated with the Bio*Me* individuals (Fig. 1), namely: glucose levels, hemoglobin A1c, total cholesterol, LDL cholesterol, HDL cholesterol, triglycerides, high sensitivity C reactive protein, diastolic blood pressure, systolic blood pressure, and white blood cell count. Before analyzing these traits in relation to LoFs, we removed trait data that was associated with ongoing medication use that potentially affected the trait (Supplementary Methods; Supplementary Results; Additional file 1: Figure S2B; Additional file 1: Figure S3-S6; Additional file 2: Table S2-S5). We required there to be at least three carriers of LoFs for a given gene with CVD-trait measurements in order to be analyzed, resulting in 1371 of the original 2143 genes examined for their association with CVD traits (see Methods). A total of 644 significant LoF-CVD trait associations involving 433 genes were identified (linear regression, *p* < 0.1) (Fig. 1; Additional file 2: Table S6). 79% of the LoFs associated with a CVD trait were genotyped. The LoF genes were both associated with higher and lower levels of the CVD traits (Additional file 1: Figure S4). The statistically most significant LoF-CVD trait association was reassuringly found in *PCSK9* associated with lower plasma LDL cholesterol (*p* = 1.1E-09, β = − 43 mg/dL) as well as plasma cholesterol (*p* = 4.3E-07, β = − 44 mg/dL; Additional file 2: Table S6). We also identified a LoF in *APOC3* that was associated with reduced levels of plasma triglycerides (*p* = 5.6E-03, β = − 60 mg/dL) as expected.

### Integrating RNA expression evidence from STARNET to the putative associations

Limitations of the Bio*Me* Biobank analysis of LoFs revolve mainly around sample size, availability of trait measurements, trait filtration, and the low frequency of most LoFs (see also Discussion). As such, many of the LoF-CVD trait associations may risk being inflated due to a skewed distribution of CVD trait levels in a few LoF carriers. At the same time, sample size issues could mask true associations trending towards significance (hence we used a less stringent cutoff *p* < 0.1). To address some of these limitations and to seek functional evidence as to what organ the LoF may impact for a given CVD trait, we sought associations between the expression levels of LoF-harboring genes and corresponding CVD traits in RNAseq data from the STARNET study [27] (Fig. 1; see Methods). Of the 644 LoF-CVD trait associations found in Bio*Me*, 241 also had at least one significant (*p* < 0.05) gene expression-CVD trait association in STARNET in at least one of the seven tissues, corresponding to 194 unique genes (Fig. 1). Out of these, 115 unique genes, 134 gene-trait associations and 165 combinations of gene-trait-tissue associations were concordant (Fig. 1; Additional file 2: Table S7), i.e. LoFs associated with lower levels of a CVD trait in Bio*Me* also showed a positive correlation with expression levels in STARNET and vice versa. In the 115 genes with concordant associations, 82% of the LoFs were genotyped. The discordant significant results between the two data sources, while not considered for follow-up evaluation, could still reflect true biological associations that could not be reliably discerned due to study limitations.

### Validation of hepatic gene targets predicted to lower plasma cholesterol and triglyceride levels in an in vitro model

**Fig. 3:**
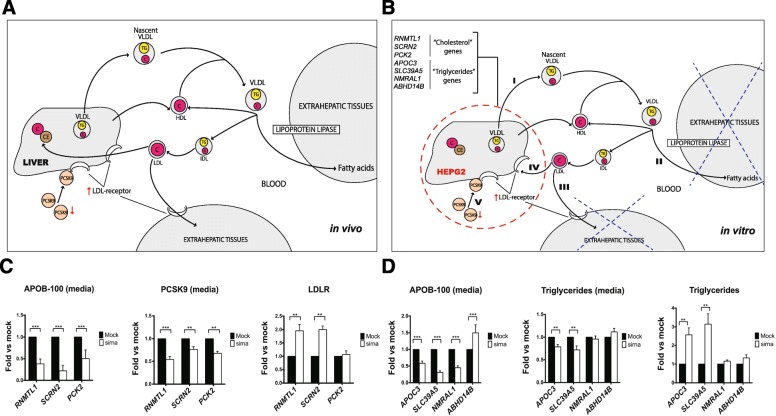
In vitro validation of candidate genes for lowering plasma cholesterol and triglycerides. **a** Schematic illustration of lipoprotein metabolism in vivo*,* and **b** An in vitro HepG2 cell model to validate three hepatic plasma cholesterol and three hepatic triglyceride candidate genes. I) Cholesterol- and triglyceride-containing very low density lipoprotein (VLDL) particles are synthesized in, and secreted from, the liver to circulation elevating plasma levels of cholesterol and triglycerides. II) The VLDL particles then travel in blood to microcirculation in peripheral tissues, such as the skeletal muscle and adipose tissue, where lipoprotein lipase (LPL) anchored to the endothelium mediates hydrolysis of VLDL-triglycerides forming free fatty acids that are taken up by the local tissue. This extra-hepatic process lowers plasma triglyceride levels. III) The LPL-mediated hydrolysis of VLDL particles results in the formation of smaller cholesterol-rich low-density lipoproteins (LDL) particles. Some LDL particles are taken up by LDL receptors in extra-hepatic tissues, a process that lowers plasma levels of LDL and cholesterol. IV) The most important regulatory process of plasma LDL and cholesterol levels is, however, the uptake of LDL by hepatic LDL receptors. V) Last, uptake of LDL by the LDL receptor in the liver is inhibited by hepatic synthesis of PCSK9 that binds to the LDL receptors and permits their recirculation away from the hepatocyte cell surface (and LDL receptor degradation) effectively lowering the uptake of LDL particles. Thus, high levels of hepatic PCKS9 lead to reduced LDL uptake and higher plasma cholesterol levels. The HepG2 in vitro model of the liver was chosen as it is the most important organ to control plasma triglyceride and cholesterol levels (**a**) and since all lipid-associated candidate genes were identified in STARNET liver RNA-seq data. However as illustrated in panels **a** and **b**, an in vitro model of the liver in the form of HepG2 cells cannot fully model lipid metabolism and the extrahepatic tissue contribution in vivo. **c** For the plasma cholesterol-lowering candidate genes (*RNMTL1*, *SCRN2* and *PCK2*), Apolipoprotein B-100 (APOB-100), Proprotein Convertase Subtilisin/Kexin type 9 (PCSK9) protein levels were measured in the cell media whereas LDL-receptor (LDLR) was measured in cell lysates. **d** For the plasma triglyceride-lowering candidate genes (*APOC3*, *SLC39A5*, *NMRAL1*, *ABHD14B*), Apolipoprotein B-100 (APOB-100), triglycerides (TG) were measured in cell media and lysates. In all experiments, the silencing efficiency of each gene resulted in more than 90% decrease in gene expression measured after 72 h incubation, including 24 h treatment with oleic acid performed before cell harvesting. Values are means ± SEM. Results are based on 3 biological replicates. *p* < 0.0332 (*), *p* < 0.0021 (**), *p <* 0.0002 (***). IDL, intermediate-density lipoprotein. Computational analysis results of the genes tested in (**c**) and (**d**) are shown in Additional file 1: Figure S9

We selected 10 genes (Table 1) associated with either lower plasma cholesterol or triglyceride levels in both Bio*Me* (β < 0, *p* < 0.1) and STARNET liver tissue (β > 0, *p* < 0.05) for further experimental validation by performing gene silencing using the HepG2 in vitro cellular model of hepatocytes. A hepatocyte model was selected since the liver is the primary organ responsible for regulating systemic cholesterol and triglyceride levels (see below). Five genes, *CYP2C19*, *PCK2*, *RNMTL1* (*MRM3*), *SCRN2*, *UGT1A4,* associated with lower levels of plasma cholesterol (Fig. 3a) were selected due to their potential role in hepatic cholesterol metabolism for in vitro validation. *PCK2* was included at our significance threshold (*p* = 0.10) and *UGT1A4 and RNMTL1* were included using lower variant imputation genotype probability scores, making in silico results from these in this analysis more speculative. Validation was performed by measuring intracellular levels of LDLR and in media, PCSK9 and APOB100 after individual silencing by siRNA in HepG2 cells (Fig. 3b). While we observed robust expression levels of *PCK2, RNMTL1,* and *SCRN2* in HepG2 cells, *CYP2C19* and *UGT1A4* were not expressed at detectable levels and were therefore excluded from the validation. For these experiments, *PCK2, RNMTL1* and *SCRN2* achieved > 90% inhibition of gene expression by siRNA (Additional file 2: Table S9). Individual silencing of *RNMTL1* and *SCRN2* both significantly reduced levels of media APOB100 and PCSK9 and concurrently increased cellular LDLR levels (Fig. 3c). Silencing of *PCK2* also reduced media levels of APOB100 and PCSK9 but had no significant effect on LDLR levels (Fig. 3c).

**Table 1:** Selection of novel genes involved in regulating cholesterol or triglyceride levels for in vitro functional studies

Gene	Description	Trait	*p*-value (Bio*Me*)	β (Bio*Me*)	*p*-value (STARNET)	β (STARNET)	Tissue (STARNET)
*CYP2C19*	Cytochrome P450 Family 2 Subfamily C Member 19	TC	0.089	−32	0.033	0.10	LIV
*PCK2*	Phosphoenolpyruvate Carboxykinase 2, Mitochondrial	TC	0.10	−23	0.00053	0.55	LIV
*RNMTL1*	RNA methyltransferase-like protein 1	TC	0.054	−36	0.016	0.60	LIV
*SCRN2*	Secernin 2	TC	0.089	−26	0.0026	0.39	LIV
*UGT1A4*	UDP Glucuronosyltransferase Family 1A4	TC	0.069	−13	2.1E-05	0.70	LIV
*ABHD14B*	Abhydrolase Domain Containing 14B	TG	0.027	−11	0.026	0.47	LIV
*APOC3*	Apolipoprotein C3	TG	0.0056	−56	0.0065	0.38	LIV
*CES3*	Carboxylesterase 3	TG	0.061	−40	0.00011	0.42	LIV
*NMRAL1*	NmrA-Like Family Domain Containing 1	TG	0.080	−28	0.045	0.28	LIV
*SLC39A5*	Solute Carrier Family 39 Member 5	TG	0.092	−70	0.0036	0.43	LIV

Negative β in Bio*Me* and positive β in STARNET indicate LoF and low gene expression are associated with decreased trait measurements

*LIV* liver, *TC* total cholesterol, *TG* triglycerides

Gene silencing in HepG2 cells was also used to confirm the role of *APOC3,* and to assess the function of three novel candidate genes (*ABHD14B*, *NMRAL1*, and *SLC39A5*) found to be associated with lower levels of plasma triglycerides in Bio*Me* and STARNET liver data. CES3 was not selected for further in vitro validation because of its well-documented effect in lowering plasma triglycerides and cholesterol levels in liver-specific KO mice [40] and in reducing the secretion of ApoB100 and triglyceride by its pharmacological inhibition in human hepatocytes [41]. As the liver has negligible or no role in regulating plasma triglyceride uptake, the roles of the three new hepatic genes *ABHD14B*, *NMRAL1*, and *SLC39A5* (if any) most likely are through facilitating hepatic secretion of very-low density lipoproteins (VLDL, Fig. 3a). *APOC3* is best known for increasing plasma triglyceride levels by interfering with lipoprotein lipase-mediated hydrolysis of plasma triglyceride-rich VLDL particles on the endothelial surface of peripheral tissues (e.g., in fat). However, recent evidence shows that hepatic APOC3 also facilitates the secretion of VLDL from the liver [42]. Silencing of *APOC3* and *SLC39A5* in HepG2 cells reduced media levels of APOB100 and triglycerides and at the same time increased intracellular levels of triglycerides, presumably due to secondary accumulation following reduced secretion of VLDL (Fig. 3d). Silencing of *NMRAL1* and *ABHD14B*, respectively, reduced and markedly increased media levels of APOB100 but had no apparent effect on levels of media or cellular triglycerides.

### A knowledge-driven approach to identify targets for in vivo validation

We also applied a knowledge-driven approach to validate potential therapeutic targets in parallel with the data-driven approach (Fig. 1). We filtered the complete list of 115 genes with concordant associations in Bio*Me* (LoF-CVD trait) and STARNET (expression-CVD trait, in any tissue) using GO annotations. A total of 90 genes (78.3%) had at least one GO-associated biological process term. Nine of these 90 genes corresponding to 11 gene-trait associations had at least one GO term related to the CVD traits of interest (e.g., biological processes relating to blood pressure, glucose, cholesterol or triglycerides) (Table 2; Additional file 2: Table S8 for GO annotation).

**Table 2:** Significant gene-trait associations (Bio*Me p* < 0.1, STARNET *p* < 0.05) of genes known to be involved in lipid and glucose metabolism based on GO annotation

	Description	GO BP annotation	Trait	*p*-value (Bio*Me*)	β (Bio*Me*)	*p*-value (STARNET)	β (STARNET)	Tissue (STARNET)
*ACOT11*	Acyl-CoA Thioesterase 11	GO:0006631 (fatty acid metabolic process)	Glucose	0.059	40	0.040	−0.28	SKLM
*ACSM3*	Acyl-CoA Synthetase Medium-Chain Family Member 3	GO:0006631 (fatty acid metabolic process)	LDL Cholesterol	0.060	−4.3	0.032	0.10	SKLM
*ADCY4*	Adenylate Cyclase 4	GO:007137 (cellular response to glucagon stimulus)	Glucose	0.0095	39	0.025	−0.40	MAM
*ADCY4*	Adenylate Cyclase 4	GO:007137 (cellular response to glucagon stimulus)	Hemoglobin A1c	0.034	1.4	0.034	−0.14	SF
*AGMO*	Alkylglycerol Monooxygenase	GO:0019432 (triglyceride biosynthetic process)	Glucose	0.094	−16	0.021	0.33	AOR
*APOC3*	Apolipoprotein C3	GO:0006641 (triglyceride metabolic process)	Triglycerides	0.0056	−56	0.0065	0.38	LIV
*DGAT2*	Diacylglycerol Acyltransferase 2	GO:0019432 (triglyceride biosynthetic process)	Glucose	0.049	−19	0.024	0.56	LIV
*DGAT2*	Diacylglycerol Acyltransferase 2	GO:0019432 (triglyceride biosynthetic process)	Cholesterol	0.078	−24	0.036	0.089	MAM
*GAA*	Glucosidase Alpha, Acid	GO:0006006 (glucose metabolic process)	HDL Cholesterol	0.039	11	0.0021	−0.21	MAM
*PCSK9*	Proprotein Convertase Subtilisin/Kexin Type 9	GO:0008203 (cholesterol metabolic process)	Glucose	0.042	−15	4.6E-05	0.65	LIV
*PLCD4*	Phospholipase C Delta 4	GO:0006629 (lipid metabolic process)	Diastolic Blood Pressure	0.030	1.9	0.027	−2.6	MAM

*AOR* aorta, *MAM* mammary artery, *LIV* liver, *SF* subcutaneous fat, *SKLM* skeletal muscle. β values represent effect size. Positive β in Bio*Me* and negative β in STARNET indicate LoF and low gene expression are associated with increased trait measurements. Negative β in Bio*Me* and positive β in STARNET indicate LoF and low gene expression are associated with decreased trait measurements

**Fig. 4:**
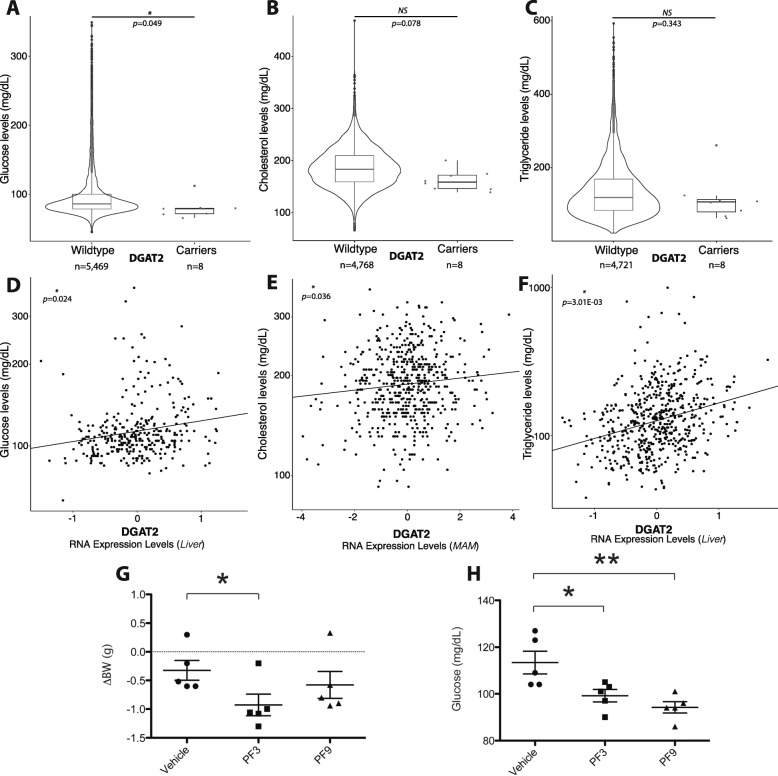
In silico analysis of DGAT2 and in vivo effect of selective DGAT1 and DGAT2 inhibitors on bodyweight and glucose levels in male C57BL/6 mice. **a** Significant association within the Bio*Me* Biobank cohort between non-carriers (*n* = 5469) and carriers (*n* = 8) of LoF *DGAT2* mutation on glucose level (logistic regression, see Methods; *p* = 0.049, β = − 19, t_β_ = − 1.4). **b** Association between non-carriers (*n* = 4768) and carriers (*n* = 8) of LoF *DGAT2* mutation on cholesterol levels (logistic regression, see Methods; *p* = 0.078, β = − 24, t_β_ = − 1.8). **c** Association between non-carriers (*n* = 4721) and carriers (*n* = 8) of LoF *DGAT2* mutation on triglyceride levels (logistic regression, see Methods; *p* = 0.34, β = − 26, t_β_ = − 1.0). **d** Association from STARNET RNA-clinical trait analysis comparing *DGAT2* expression levels with glucose levels in liver tissue (*p* = 0.024, β = 0.56). **e** Association from STARNET RNA-clinical trait analysis comparing *DGAT2* expression levels with cholesterol levels in mammary artery (*p* = 0.036, β = 0.089). **f** Association from STARNET RNA-clinical trait analysis comparing *DGAT2* expression levels with triglyceride levels in liver tissue (*p* = 0.0030, β = 0.27). **g** Body weight loss (ΔBW) of chow-fed mice treated for 3 days with vehicle, DGAT1 inhibitor (PF3), and DGAT2 inhibitor (PF9). **h** Short fasting blood glucose of chow-fed mice treated for 3 days with vehicle, DGAT1 inhibitor (PF3), and DGAT2 inhibitor (PF9) (*n* = 5). *p* < 0.05 (*), *p* < 0.01 (**), versus vehicle (student t-test)

Of these, we selected *DGAT2* for follow-up validation for several reasons. First, the *DGAT*2 expression-CVD trait association for glucose levels was identified in liver (Table 2), a tissue type with more biological relevance to the trait. Second, pharmacological inhibitors of DGAT2 are available (Futatsugi et al. 2015) for further experimental validation. Most importantly, a homolog of *DGAT2*, *DGAT1*, has been extensively pursued as a therapeutic target for obesity and diabetes yet with limited clinical success (see below), and we wanted to explore if *DGAT2* would be a more viable target. *DGAT2* exhibited significant associations with plasma cholesterol and glucose levels in both Bio*Me* and STARNET (Table 2). In the Bio*Me* Biobank, we identified a stop-gain LoF based on genotyping data (stop codon Y285*, 0.001% allele frequency) found in 12 heterozygous individuals located in the middle of the enzymatic domain of *DGAT2* (Additional file 1: Figure S7A) that likely triggers non-sense mediated decay resulting in no protein synthesis (Additional file 1: Figure S7B and S7C). Individuals with this LoF had significantly (*p* < 0.1) lower plasma glucose (Fig. 4a) and cholesterol (Fig. 4b) levels and statistically non-significant (*p* > 0.1) lower plasma triglycerides (Fig. 4c). Corresponding positive and significant (*p* < 0.05) associations with plasma levels of glucose, cholesterol and triglycerides were also observed for hepatic *DGAT2* expression in STARNET (Fig. 4d-f).

DGAT catalyzes the final step in triglyceride synthesis [43] and there are two known DGAT enzymes, DGAT1 and DGAT2. DGAT1 has been extensively investigated as a therapeutic target for obesity and obesity-related metabolic disorders such as insulin resistance. Across several preclinical studies [37, 44, 45], DGAT1 inhibitors were shown to reduce body weight and improve insulin sensitivity. In contrast, DGAT2 inhibition has not been extensively investigated. Among the limited studies performed so far, antisense oligonucleotides against *DGAT2* lowered hepatic lipid levels in mice [46–48], however, pharmacological inhibition of DGAT2 with selective small molecule inhibitors has not been reported.

To evaluate the effect of specific inhibition of DGAT2 and to compare this with inhibition of DGAT1, we treated C57BL/6 mice with a highly selective DGAT1 small molecule inhibitor PF-04620110 (PF3) [49] or a highly selective DGAT2 inhibitor PF-06424439 (PF9) [38]. Treatment with the DGAT1 inhibitor was associated with significantly decreased body weight (Fig. 4g) and fasting glucose levels (Fig. 4h), consistent with previous reports [37, 44]. Similar effects were observed in mice treated with the DGAT2 inhibitor (Fig. 4g and h). However, whereas weight loss following DGAT2 inhibition was borderline significant (Fig. 4g), the lowering of fasting plasma glucose levels was more pronounced than in mice treated with the DGAT1 inhibitor (Fig. 4h).

## Discussion

In drug discovery and development, half of all therapeutics fail in phase 2 clinical trials due to lack of efficacy [50]. This is immensely expensive and pharmaceutical companies are urgently looking for new strategies to better select targets at an earlier stage. In this regard, the power and accuracy of human genetics for target selection is increasingly being recognized [11]. In the current study, we identified and cataloged 2818 LoFs in 2143 genes from genotyping and the imputed data in the Mount Sinai Bio*Me* Biobank [51]. LoFs in 433 of these genes were found to be associated with at least one of 10 major CVD traits in clinical data of the MHS-EMRs after careful annotation. 115 genes were further confirmed as being concordantly associated with the same trait in the world’s largest human RNAseq datasets from blood, vascular, and metabolic tissues in STARNET [27]. Although the genetic data in Bio*Me* is based on genotyping and imputation, the majority (75%) of the analyzed 2818 LoFs and 82% of the LoFs in the 115 genes with concordant associations were genotyped, enhancing our confidence in the results. Using complementary data- and knowledge-driven approaches, we then successfully validated five novel liver genes for lowering of plasma cholesterol and triglycerides in HepG2 cells. In addition, we revealed that DGAT2 inhibitors have marked effects on plasma glucose in vivo. This represents the first systematic study of a large, hospital-based EMR-linked biobank to uncover and catalog novel LoFs associated with CVD traits that were then validated in global gene expression data. As such, it serves to improve our understanding of CVD biology and highlights novel putative CVD target genes that merit further investigation.

In comparison to the DiscovEHR study [19] where 176,000 LoFs were identified from 50,726 exomes, we identified significantly fewer LoFs in the Bio*Me* cohort. Besides a smaller sample size and different makeup of ethnicity, the reason for the large discrepancy in predicted number of LoFs between the studies is that we used genotyping and the imputed data. Nearly 60% of the LoFs detected in the DiscovEHR cohort are singletons and 98.5% had an allele frequency of < 0.1%. With respect to associations between LoFs and clinical traits, the DiscovEHR study only focused on nine therapeutic targets with drugs approved by the FDA or in clinical trials. In contrast, we validated novel gene-trait associations in independent gene expression data and carried out in vitro and in vivo functional studies.

We identified six novel candidate CVD-related genes using our pipeline. For these six genes we found strong support for their roles in hepatic control of plasma cholesterol (*PCK2*, *RNMTL1* and *SCRN2*) and triglyceride (*ABHD14B*, *NMRAL1* and *SLC39A5)* levels using gene silencing in HepG2 cells (Fig. 3). None of the three plasma cholesterol-lowering candidate genes have previously been implicated in hepatic cholesterol homeostasis (see Supplementary Discussion). Given the pronounced and concordant effects of silencing *PCK2*, *RNMTL1*, and *SCRN2* in reducing media levels of APOB100 and PCSK9 and increasing the expression of LDLR in HepG2 cells (all but PCK2) these genes should be further evaluated as novel targets for lowering plasma cholesterol.

LoFs in three previously unassociated genes (*ABDH14B, NMRAL1* and *SLC39A5*), the documented plasma triglyceride-related genes, *APOC3* [52, 53] and CES3 [40, 41] were found to be associated with lower plasma triglyceride levels in Bio*Me* and STARNET liver data. Again using gene silencing in HepG2 cells (Fig. 3), we not only found strong support for this role of *APOC3* but also equally for *SLC39A5,* with concurrent and marked effects on levels of both media and intracellular triglycerides and media APOB100 (Fig. 3d). Silencing of *ABDH14B and NMRAL1* affected APOB100 secretion in opposite ways but had no effect on either media or cellular triglycerides. Taken together, our validation confirms the role of *APOC3*, and strongly suggests that *SLC39A5* may be an interesting candidate gene for lowering plasma triglyceride levels. For *NMRAL1* and *ABDHD14B,* marked and opposite effect were observed on media APOB100 whereas media and cellular triglyceride levels were unaffected. Thus the roles of these two genes require further scrutiny.

Our study also provided human genetic support for DGAT2 as a therapeutic target, which has not previously been reported. Although both DGAT1 and DGAT2 catalyze the same reaction in the last step of triglyceride synthesis, often in the same cells, DGAT1 but not DGAT2 has been pursued for more than a decade as a therapeutic target for obesity and metabolic disorders such as insulin resistance. This is primarily because *DGAT1* mouse knockout (KO) models are resistant to diet-induced obesity, show decreased levels of tissue triglycerides, and have increased sensitivity to insulin and leptin [54, 55]. In contrast, *DGAT2* KO mice die soon after birth, apparently from profound reductions in energy metabolism substrates and impaired skin permeability [56]. Although DGAT1 inhibitors consistently demonstrate metabolic benefit in various animal models [37, 44, 45], results from several clinical trials have been disappointing [57]. Specifically, while most of the clinical candidates exhibited the desired pharmacokinetic properties and target engagement, they failed to show effects on GLP-1 levels and insulin sensitivity. Moreover, there were gastrointestinal side effects such as nausea, diarrhea, and vomiting [57, 58]. Indeed, it was later discovered that a rare LoF splice site mutation in *DGAT1* in an Ashkenazi Jewish family is linked to a congenital diarrheal disorder (CDD) [59]. Therefore, the lack of success from inhibiting DGAT1 in humans, despite promising results from animal models, may be explained by the differing phenotypes of *DGAT1* deficiency in human and mouse, again emphasizing the significance of target validation using human genetics. Association of LoF mutations or reduced expression in *DGAT2* with reduced plasma cholesterol and glucose levels in both Bio*Me* and STARNET cohorts in this study, coupled with the observation that a selective DGAT2 inhibitor had similar effects in mice as DGAT1 inhibitors (Fig. 4), suggests that DGAT2 could be a more preferable therapeutic target in humans than DGAT1.

We recognize there are several limitations to our study. First, as noted above, biobank data is not generated based upon on a prospective design for specific diseases or disease-relevant traits, hence clinical data quality is a concern. Even though we developed a thorough and individualized QC process for the 10 analyzed traits, with manual curation by two cardiologists to compile a list of medications and their related diseases that may affect each trait, there could be other factors affecting trait measurements that were not accounted for. These may include unreported medication use or pathophysiologic conditions when the measurements were taken. The sample size is another limitation with biobank data. While we analyzed associations of LoF genotypes with 10 CVD and metabolic traits, we also attempted to perform similar association analysis on risk for CVDs. However, due to small sample sizes for each specific disease, the analysis did not yield interpretable results and is thus omitted from this report. Second, computational prediction of LoFs is still an empiric, imperfect process. Without experimental confirmation of each predicted LoF, there may be false positive and false negative associations for each gene. It is conceivable that some missense mutations lead to diminished or complete loss of protein function, but they are not included in our analysis. This is potentially problematic because LoFs are relatively rare in the human genome, and any false positive or false negative annotations may significantly impact the sample size of the case group (i.e. LoF carriers), causing identification of spurious LoF-trait associations. Third, genetic associations with diseases or disease related traits are often dependent upon racial and ethnic background. While the Bio*Me* Biobank is racially diverse, the STARNET patients are predominantly White Caucasians. Thus, lack of validation in the STARNET cohort does not necessarily indicate the LoF/CVD-trait associations identified the Bio*Me* Biobank cohort are false. However, genes with concordant CVD-trait associations in both Bio*Me* Biobank and STARNET merit further scrutiny. In addition, due to the relatively low frequency of many of the variants characterized in this study coupled with the sample size of the Bio*Me* Biobank cohort, we were underpowered to perform any form of stratified analysis to assess the contribution of racial or ethnic background to these associations. Lastly, we elected to use uncorrected, lenient *p*-value thresholds for both Bio*Me* and STARNET data analyses (*p* < 0.1 and *p* < 0.05, respectively) with the understanding that we were less concerned with specific individual associations but more with those that were orthogonally significant in multiple datasets and validated experimentally.

## Conclusion

In conclusion by leveraging the unique EMR-linked Bio*Me* Biobank, we were able to identify and catalog 433 LoF-harboring genes found to be associated with at least one of 10 major CVD traits. Next and in contrast to recently published genotype-phenotype association studies [15, 19, 60], we successfully validated 115 of these putative gene-CVD associations using STARNET RNA-seq data from seven metabolic and vascular tissues. As a result, experimental in vitro validations of *PCK2*, *RNMTL1* and *SCRN2,* and *ABHD14B*, *NMRAL1* and *SLC39A* confirmed roles for these genes in regulating plasma levels of cholesterol and triglycerides, respectively. Moreover, hepatic DGAT2 merits further attention as a parallel, or possibly alternative, drug target to DGAT1 for improving insulin resistance and reducing body weight.

## Additional files

Additional file 1: Supplementary Material, Supplementary Methods, Supplementary Results, Supplementary Discussion, Supplementary Figure legends, Supplementary Table legends; **Figures S1-S9.** (PDF 8049 kb)

Additional file 2: **Tables S1-S9.** (XLSX 1175 kb)

## Data Availability

Access to data in Bio*Me* and STARNET can be achieved through formal request using the database of Genotypes and Phenotypes (dbGaP) using study accessions phs000925.v1.p1 and phs001203.v1.p1 respectively.

## Abbreviations

CVD: Cardiovascular disease
EMR: Electronic medical record
HDL: High-density lipoprotein
LDL: Low-density lipoprotein
LoF: Loss of function
