# Correction to: Integrative analysis of loss-of-function variants in clinical and genomic data reveals novel genes associated with cardiovascular traits

**DOI:** 10.1186/s12920-019-0573-9

**Published:** 2019-11-05

**Authors:** Benjamin S. Glicksberg, Letizia Amadori, Nicholas K. Akers, Katyayani Sukhavasi, Oscar Franzén, Li Li, Gillian M. Belbin, Kristin L. Ayers, Khader Shameer, Marcus A. Badgeley, Kipp W. Johnson, Ben Readhead, Bruce J. Darrow, Eimear E. Kenny, Christer Betsholtz, Raili Ermel, Josefin Skogsberg, Arno Ruusalepp, Eric E. Schadt, Joel T. Dudley, Hongxia Ren, Jason C. Kovacic, Chiara Giannarelli, Shuyu D. Li, Johan L. M. Björkegren, Rong Chen

**Affiliations:** 1 Department of Genetics and Genomic Sciences, The Icahn Institute for Genomics and Multiscale Biology, Icahn School of Medicine at Mount Sinai, One Gustave L. Levy Place, New York, NY 10029 USA; 2 The Institute for Next Generation Healthcare, Icahn School of Medicine at Mount Sinai, One Gustave L. Levy Place, New York, NY 10029 USA; 3 Cardiovascular Research Center and Cardiovascular Institute, Icahn School of Medicine at Mount Sinai, One Gustave L. Levy Place, New York, NY 10029 USA; 4 Department of Pathophysiology, Institute of Biomedicine and Translation Medicine, University of Tartu, Biomeedikum, Ravila 19, 50411 Tartu, Estonia; 5 Clinical Gene Networks AB, Jungfrugatan 10, 114 44 Stockholm, Sweden; 6 Charles Bronfman Institute of Personalized Medicine, Icahn School of Medicine at Mount Sinai, One Gustave L. Levy Place, New York, NY 10029 USA; 7 Sema4, a Mount Sinai venture, Stamford, CT 06902 USA; 8 Department of Preventive Medicine, Icahn School of Medicine at Mount Sinai, One Gustave L. Levy Place, New York, NY 10029 USA; 9 Department of Immunology, Genetics and Pathology, Uppsala University, 751 85 Uppsala, Sweden; 10 Department of Cardiac Surgery, Tartu University Hospital, 1a Ludwig Puusepa Street, 50406 Tartu, Estonia; 11 Integrated Cardio Metabolic Centre, Department of Medicine, Karolinska Institutet, Karolinska Universitetssjukhuset Huddinge, 141 86 Stockholm, Sweden; 12 Department of Health Policy and Research, Icahn School of Medicine at Mount Sinai, One Gustave L. Levy Place, New York, NY 10029 USA; 13 Department of Pediatrics, Herman B Wells Center for PediatricResearch, Center for Diabetes and Metabolic Diseases, Stark Neurosciences Research Institute, Indiana University, 635 Barnhill Dr., MS2049, Indianapolis, IN 46202 USA; 14 Bakar Computational Health Sciences Institute, University of California San Francisco, San Francisco, CA 94158 USA; 15 Integrated Cardio Metabolic Centre, Department of Medicine, Karolinska Institutet, Novum, 14157 Huddinge, Sweden

**Correction to: BMC Med Genomics**

**https://doi.org/10.1186/s12920-019-0542-3**

Correction

After publication of this supplement article [1], it was brought to our attention that the eighth author’s name was incorrectly spelt. The correct name should be Kristin L. Ayers.
