# Supplementary material for: Integrative analysis of loss-of-function variants in clinical and genomic data reveals novel genes associated with cardiovascular traits
Source: BMC Med Genomics. 2019 Jul 25;12(Suppl 6):108. doi: 10.1186/s12920-019-0542-3 (PMC6657044; doi:10.1186/s12920-019-0542-3)
Supplement: Supplementary file 1 — Supplementary Material, Supplementary Methods, Supplementary Results, Supplementary Discussion, Supplementary Figure legends, Supplementary Table legends; Figures S1-S9. (PDF 8049 kb) [file 12920_2019_542_MOESM1_ESM.pdf]

Integrative analysis of loss-of-function variants in clinical and genomic data reveals novel genes associated with cardiovascular traits

Benjamin S. Glicksberg[1,2], Letizia Amadori[1,3], Nicholas K. Akers[1], Katyayani Sukhavasi[4], Oscar Franzén[1,5], Li Li[1,2], Gillian M. Belbin[1,6], Kristin L. Akers[1,7], Khader Shameer[1,2], Marcus A. Badgeley[1,2], Kipp W. Johnson[1,2], Ben Readhead[1,2], Bruce J. Darrow[3], Eimear E. Kenny[6,8], Christer Betsholtz[9], Raili Ermel[10], Josefin Skogsberg[11], Arno Ruusalepp[5,9], Eric E. Schadt[1,2,5,7], Joel T. Dudley[1,2,12], Hongxia Ren[13], Jason C. Kovacic[3], Chiara Giannareli[1,3], Shuyu D. Li[1,7*], Johan L. M. Björkegren[1,4,5,11*], and Rong Chen[1,7*]

[1] Department of Genetics and Genomic Sciences, The Icahn Institute for Genomics and Multiscale Biology, Icahn School of Medicine at Mount Sinai, One Gustave L. Levy Place, New York, NY 10029, USA.
[2] The Institute for Next Generation Healthcare, Icahn School of Medicine at Mount Sinai, One Gustave L. Levy Place, New York, NY 10029, USA.
[3] Cardiovascular Research Center and Cardiovascular Institute, Icahn School of Medicine at Mount Sinai, One Gustave L. Levy Place, New York, NY 10029, USA.
[4] Department of Pathophysiology, Institute of Biomedicine and Translation Medicine, University of Tartu, Biomeedikum, Ravila 19, 50411, Tartu, Estonia.
[5] Clinical Gene Networks AB, Jungfrugatan 10, 114 44 Stockholm, Sweden.
[6] Charles Bronfman Institute of Personalized Medicine, Icahn School of Medicine at Mount Sinai, One Gustave L. Levy Place, New York, NY 10029, USA.
[7] Sema4, a Mount Sinai venture, Stamford, CT 06902, USA
[8] Department of Preventive Medicine, Icahn School of Medicine at Mount Sinai, One Gustave L. Levy Place, New York, NY 10029, USA.
[9] Department of Immunology, Genetics and Pathology, Uppsala University, 751 85 Uppsala, Sweden.
[10] Department of Cardiac Surgery, Tartu University Hospital, 1a Ludwig Puusepa Street, 50406 Tartu, Estonia.
[11] Integrated Cardio Metabolic Centre, Department of Medicine, Karolinska Institutet, Karolinska Universitetssjukhuset Huddinge, 141 86 Stockholm Sweden.
[12] Department of Health Policy and Research, Icahn School of Medicine at Mount Sinai, One Gustave L. Levy Place, New York, NY 10029, USA.
[13] Department of Pediatrics, Herman B Wells Center for Pediatric Research, Center for Diabetes and Metabolic Diseases, Stark Neurosciences Research Institute, Indiana University, 635 Barnhill Dr., MS2049, Indianapolis, IN 46202, USA.

* To whom correspondence should be addressed: Rong Chen, Email: rong.chen@mssm.edu, Shuyu D. Li, Email: shuyudan.li@mssm.edu, and Johan L. M. Björkegren. Email: johan.bjorkegren@mssm.edu.

SUPPLEMENTARY METHODS

Overview of Annotation of LoF variants

Genotyping and subsequent imputation yielded data for more than 37 million genetic variants in the Bio*Me* Biobank cohort. There are a number of tools available to annotate effects of mutations on gene function and previous related studies have used various tools for their annotation procedures. As different annotators have their strengths and weaknesses, we decided to utilize three widely-utilized annotators to identify the predicted effects of these mutations: VAT [1], ANNOVAR [2] and SnpEff [3]. Following the example of previous studies, we limited LoF annotation to “stop gain”, “splice site”, and “frame shift” only with “high” or “full” impact. Notably, the proportion of three types of the predicted LoF variants varied with different annotators (Supplementary Fig. 1A), reflecting some degree of discrepancy and the possibility of both false positive and false negative calls by each annotator. We then empirically defined LoF as a variant that was annotated so in at least two out of the three annotators (i.e. a consensus annotation) (Supplementary Fig. 1B). After further QC steps (see Methods; Supplementary Fig. 2A), we ended up with 2,818, high confidence variants collapsed to 2,143 genes. All identified LoF variants along with their annotations and frequencies in the Bio*Me* Biobank cohort and public datasets are presented in Supplementary Table 1. There are 10 variants that are not seen in these public datasets, but they were directly measured on the exome chip.

Determining genetic ancestry

We utilized Principal Component Analysis (PCA) for dimensionality reduction on genetic data of the cohort to obtain a metric for genetic ancestry. We performed the following QC steps on the genotype data: we cleaned the data for individual and site level missingness to a threshold of 95%, kept only common variants that had >=1% minor allele frequency, thinned linkage disequilibrium to a r^2 of 0.3, and removed human leukocyte antigen and lactase genetic regions. We ran this cleaned genotype data into the EIGENSOFT [4] smartpca tool to generate PCs for each individual by first training the space on the European ancestry individuals from Utah (CEU), Yoruba in Ibidan, Nigeria (YRI), and Han Chinese in Beijing, China (CHB), which were used as European, African, and East Asian ancestry reference panels from 1,000 genomes project, respectively [5]. For the models of our analyses, we used the top five PCs for each individual as covariates.

Trait measurement processing, quality control, and confounding factor filtering

The 10 metabolic/cardiovascular-related traits selected for analysis, specifically: glucose, hemoglobin A1c, total cholesterol, LDL cholesterol, HDL cholesterol, triglycerides, high sensitivity C reactive protein, diastolic blood pressure, systolic blood pressure, and white blood cell count, were collected from the MSH EMR databases, in the form of individual, value, date, and unit of measurement. As such, an individual could have multiple associated measurements per trait. As mentioned, these trait values are susceptible to confounding factors, such as disease status, medication use, and infrastructural factors of the EMR database. As no easily adaptable, universal procedure exists to address these factors, we devised and performed a rigorous quality control procedure per trait.

Furthermore, while there are some standardized procedures that exist for these purposes, such as adding 15 and 10 mmHg to systolic and diastolic measurements respectively to patients taking a blood pressure lowering medication [6], we opted to perform identical quality control procedures for each trait for a few reasons: first, our filtering methodology is more conservative; second, it is more comprehensive (including many potentially relevant medications); third, it follows the same rules for all traits, enhancing our ability to compare associations across traits; and lastly, it makes no assumptions on effect of drug (i.e. blood pressure medications might affect individuals differently, which is masked by a standard adjustment procedure). As an initial step, we only included measurements that were taken during an “Outpatient” encounter, excluding both “Inpatient” and “Ambulatory/Emergency” encounters as the latter are more likely to represent active disease states.

Excluding relevant medication-affected trait values criteria and procedure. We utilized medication prescriptions as markers of an affected measurement (to be excluded) as medications can both indicate disease presence and affect trait levels independently. We incorporated medication data for any type of patient visit (Inpatient, Outpatient, or Ambulatory/Emergency) to gain a complete picture of patient health state. Each prescription instance is comprised of drug name, dosage, route of administration (e.g. oral), and visit type. The medication data retrieved from the EMR is directly connected to the EPIC system, which utilizes First DataBank (First Databank, South San Francisco, CA; http://www.fdbhealth.com) in its back end to manage drug-related data. The First DataBank hierarchical framework provides ontological mappings for all medications to a specific Therapeutic class, Pharmaceutical class, and Pharmaceutical sub-class, which is particularly useful as a single medication can have a multitude of string identifiers (i.e. different dosages, generic vs. brand names). Accordingly, we connected the drug name within each prescription instance to First DataBank mappings. The First DataBank contains information on 115,677 medications, 48 Therapeutic classes, 885 Pharmaceutical classes, and 1,577 Pharmaceutical sub-classes.

Two physician cardiologists were in charge of identifying specific classes of medications that could affect each trait (Supplementary Table 2). If there was any disagreement, a third expert made the final decision. As such, we compiled a list of all medications that can affect each trait through mapping from Therapeutic class. Additionally, we identified classes of medications that can affect all traits of interest (e.g. cancer treatment medication). To enact this filtration procedure, we compared dates of measurement collection to dates of drug prescription. If an associated medication was prescribed three months before or three months after (180 day total window) a measurement was taken, we flagged it for removal (see Supplementary Fig. 2B for a visual description of this filtering procedure). The three month window was selected as a typical timeline for which a prescription refill would be necessary. As mentioned, we also included a window to exclude the measurement if a relevant medication was prescribed within three months after the collection date. This was enacted to safeguard against latent or undiagnosed disease states that could still affect the trait level during pathogenesis.

Trait measurement exclusion criteria and procedure. We first filtered trait data for mismatched units. However, clerical errors (i.e. incorrect measurement label or typos) could still allow physiologically

impossible measurements to be in our dataset. Accordingly, we first compiled normal reference ranges per trait and then filtered values that were: greater than 3x the upper reference range and lower than 1/3 the lower reference range. We present the filtering figures due to outliers in Supplementary Table 4.

Once all incorrectly labeled, outlier, and medication/disease-affected measurements were filtered out, we combined any remaining measurements into a single median value per person. Any individuals without any remaining clean measurements were excluded from the particular trait analysis. We present a list of the number of individuals and affected measurements that were excluded per trait for drug filtering along with the total number of individuals and measurements that were excluded due to drugs and outliers in Supplementary Table 3 and 4 respectively. We compile the total number of measurements and individuals used before and after these filtration steps per trait in Supplementary Table 5.

Gene ontology annotation

In order to identify knowledge-based associations of interest as further refined candidates for therapeutic validation, we intersected the combined significant associations between both cohorts with relevant Gene Ontology (GO) terms [7] (Supplementary Table 8). Specifically, we highlighted any association in which the gene had a related “Biological Process” to the traits of interest (e.g. lipid metabolic process, regulation of blood pressure).

SUPPLEMENTARY RESULTS

Effect of medication on gene-trait association results

In total, 10,072 (95.8%) individuals in the utilized cohort had at least one prescription instance, which encompasses drug name, dosage, and route of administration. On average, individuals in our cohort had 132.5 ± 264.4 (std) prescription instances. If limited to unique prescription instance (i.e. drug name-dosage-route of administration combination), individuals in our cohort had an average of 50.6 ± 58.5 (std) prescription instances. The distribution of prescriptions per individual (Supplementary Fig. 3) reflects the imperative need for this type of filtering.

To demonstrate the importance and utility of the rigorous quality control process for trait data, we performed the same analysis on the raw trait data prior to removal of medication/disease-affected measurements. While the overall numbers for each condition seem similar, the differences reveal the importance for this correction (Supplementary Fig. 5). In cases where an association was significant in the raw data but not in the cleaned data, these can be interpreted as potential false positives. In total, we found 136 instances of these potential false positive associations (*p-clean*>0.05, *p-raw*<0.05). Associations that were significant in the cleaned data but not in the raw can be interpreted as potential false negatives. We found 145 instances of potential false negatives (*p-clean*<0.05, *p-raw*->0.05). To illustrate these points, we present an example of the association between *CPA1* gene and

total cholesterol levels (Supplementary Fig. 6). Using the raw data, we identified a significant association between LoF in the *CPA1* gene and reduced cholesterol level (*p*=0.019; Supplementary Fig. 6A). However, the association is no longer statistically significant after the data was corrected by removing medication-affected measurements (*p*=0.36; Supplementary Fig. 6B). Therefore this association represents a potential false positive result.

SUPPLEMENTARY DISCUSSION

Novel genes implicated in cholesterol and triglyceride homeostasis

RNMTL1 (RNA methyltransferase-like protein 1) is a member of the RNA methyltransferase family responsible for methylation of G (1370) of the human 16S rRNA complex [8]. Inactivation of *RNMTL1* in HeLa cells by RNA interference resulted in respiratory inefficiency due to reduced mitochondrial translation [9]. In humans, *RNMTL1* is specifically expressed in liver [10]. In contrast, *SCRN2* (secernin-2) is ubiquitously expressed across many tissues and is involved in exocytosis in mast cells [11]. Other than that, *SCRN2* in muscle cells has been negatively correlated with plasma triglyceride levels in F2 mice [12]. *PCK2* (phosphoenolpyruvate carboxykinase 2) is a mitochondrial gene also expressed in a variety of human tissues (mainly in liver, kidney, pancreas, intestine and fibroblasts). It has been implicated in glucose homeostasis in the liver (in its cytosolic form) [13].

SLC39A5 (solute carrier family 39 member 5) belongs to the ZIP family of zinc transporters and plays a crucial role in controlling intracellular zinc levels [14]. It is expressed in a variety of tissues including liver, kidney, pancreas, small intestine and colon [15]. In humans, mutations in this gene have been associated with autosomal dominant myopia [16] but its functional role requires further characterization. *ABHD14B* (abhydrolase domain-containing protein 14B) is also ubiquitously expressed across tissues [17], encoding an enzyme with largely unknown function. However, some evidence suggests it is involved in nuclear transcription activation [18]. Moreover, a genetic mutation in *ABDH14B* has been associated with a mitochondrial complex III enzyme deficiency [19]. *NMRAL1* (NmrA like redox sensor 1) encodes a sensor protein that preferentially binds to NADPH. Association with argininosuccinate synthase (AS) impairs its activity and reduces the production of nitric oxide, which subsequently prevents apoptosis [20]. The encoded protein has also been shown to negatively regulate NF-kappaB in an ubiquitylation-dependent manner [21, 22].

SUPPLEMENTARY TABLE AND FIGURES LEGENDS

Supplementary Table 1. All predicted LoF mutations along with the predicted effects by annotator and frequencies within the Bio*Me* Biobank cohort and public data sources, namely 1,000 genomes [23], ExAC [24], and GO ESP6500 [25].

Supplementary Table 2. A detailed map of medication categories selected for filtration for each trait. The therapeutic and pharmaceutical classes and pharmaceutical sub-class of drugs classified by First DataBank along with the traits determined as affected by type. Also provided are the number of unique medications encompassed by each type along with the number of unique individuals in the Bio*Me* Biobank cohort that are prescribed at least one of them.

Supplementary Table 3. The trait filtering procedure due to medications. Shown are the numbers of individuals and measurements before and after medication filtration as well as the number of each that were excluded due to this step.

Supplementary Table 4. The trait filtering procedure due to biologically relevant outliers. For each trait, the lower and upper bond reference range along with the outlier cut-offs are provided. Further outlined are the numbers of individuals and measurements before and after filtration as well as the numbers of each that were excluded from this step.

Supplementary Table 5. The overall trait statistics due to both medication and outlier filtration. Shown are the numbers of individuals and measurements before and after filtration along with the number of each that were excluded due to filtering.

Supplementary Table 6. Detailed results of gene-trait association analysis in the Bio*Me* Biobank cohort.

Supplementary Table 7. Detailed results of significant associations with matching directions of effect in both Bio*Me* Biobank and STARNET cohorts.

Supplementary Table 8. Associations significant in both Bio*Me* Biobank and STARNET cohorts involved in relevant GO-related Biological Processes.

Supplementary Table 9. siRNA and Taqman assays and the degree of siRNA inhibition. siRNA and TaqMan assays used to silence genes and measure the degree of gene silencing of HepG2 cells.

Supplementary Figure 1. The predicted LoF variants in the Bio*Me* Biobank cohort. (A) Breakdown of annotation type (stop-gain, splice site, or frame shift) calls per annotation for all available variants. (B) Raw numbers and overlap of LoF annotation per annotator. A variant was deemed LoF if at least 2 out of 3 annotators called it such (overlapping sections; bolded numbers).

Supplementary Figure 2. Further details of the curation process for genetic and clinical data displayed in Figure 1. (A) The number of variants removed in the various filtering steps in the QC process leading to the final count (*n*=2,818) used for the study. (B) How measurements were filtered due to medication effects, per trait. We flagged any trait measurement that occurred either 90 days before (i.e. window of disease pathogenesis process) or after (i.e. biased due to effects of the medication) a medication prescription. The median of the remaining "clean" measurements was used as the representative value.

Supplementary Figure 3. Distribution of medication prescriptions within Bio*Me* cohort. The distribution of the (log10) number of selected medications taken per individual in the Bio*Me* BioBank in terms of all prescriptions (Overall) and unique medications (Unique).

Supplementary Figure 4. Distribution of t-statistic beta values within Bio*Me* associations. The distribution of t-statistic$_{\beta}$ values (β divided by standard error), for all nominally significant LoF gene-trait associations (p<0.05) in Bio*Me* Biobank, separated by trait.

Supplementary Figure 5. The effect of medications on gene-trait associations. The relative amount of significant gene association that overlaps (both) and is unique to cleaned trait data (i.e. medication-effects removed) and raw data (outlier filtration only), as shown for each trait.

Supplementary Figure 6. Association between *CPA1* LoF and cholesterol levels with and without controlling for medication effects. (A) Significant association in raw trait analysis (no medication filtering; p=0.019). (B) Non-significant association (p=0.36) after controlling for medications that affect cholesterol levels.

Supplementary Figure 7. The predicted LoF mutation in *DGAT2* and its predicted effect on protein structure. (A) The position of the LoF mutation (p.Tyr285*) within *DGAT2*. (B) The canonical protein structure of wild type DGAT2 protein. (C) The predicted structural effect with the LoF mutation.

Supplementary Figure 8. Effect of Box-Cox transformation on all traits in Bio*Me*/EMR cohort. (A) Untransformed trait distributions. (B) Transformed trait distribution. (C) Untransformed trait QQ plots. (D) Transformed QQ plots.

Supplementary Figure 9. Associations between LoF in the selected genes for *in vitro* validation (Fig. 3) and total cholesterol level (A-C), or triglyceride level (D-G) in Bio*Me*.

Supplementary Figure 1.

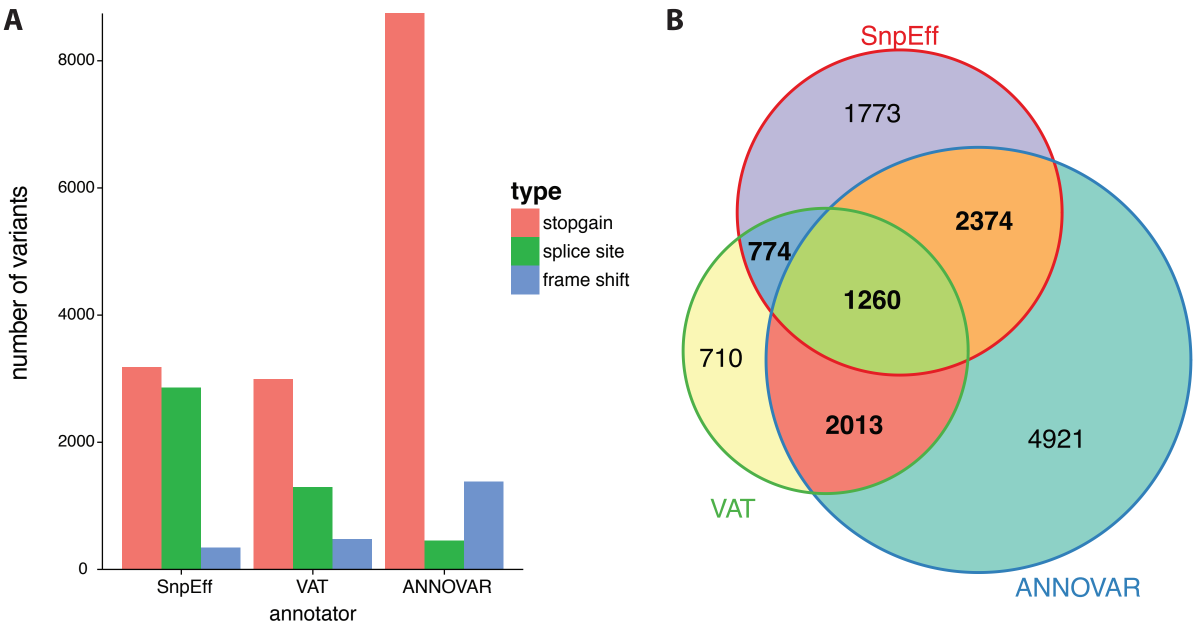

Supplementary Figure 2.

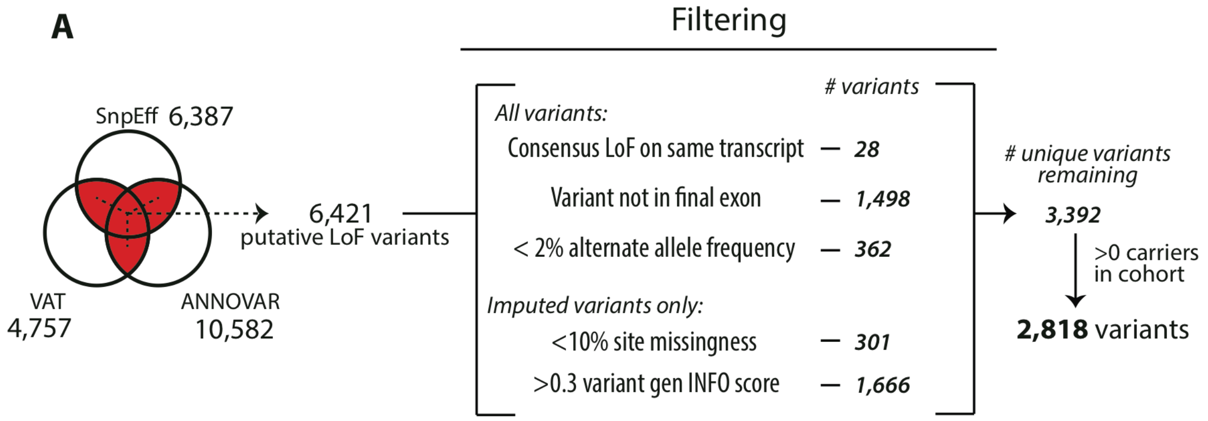

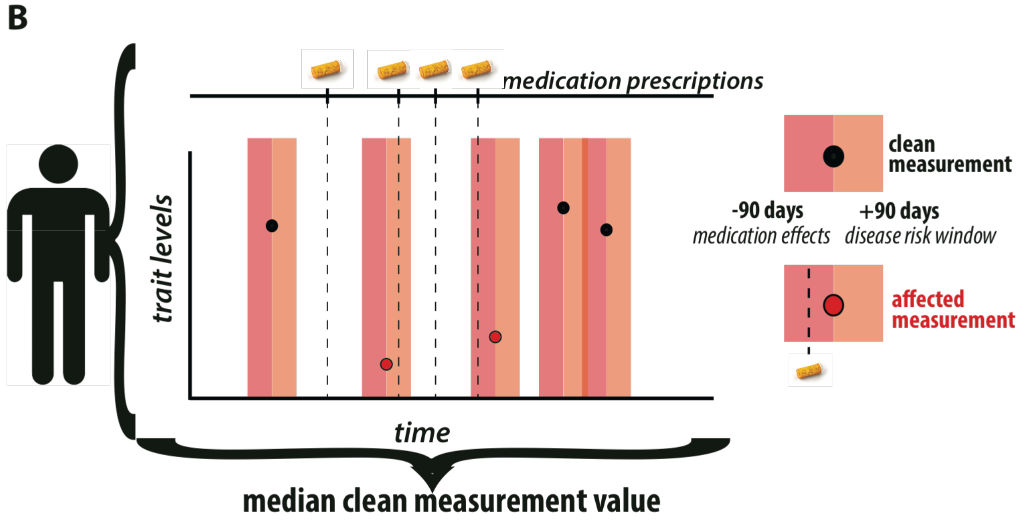

Supplementary Figure 3.

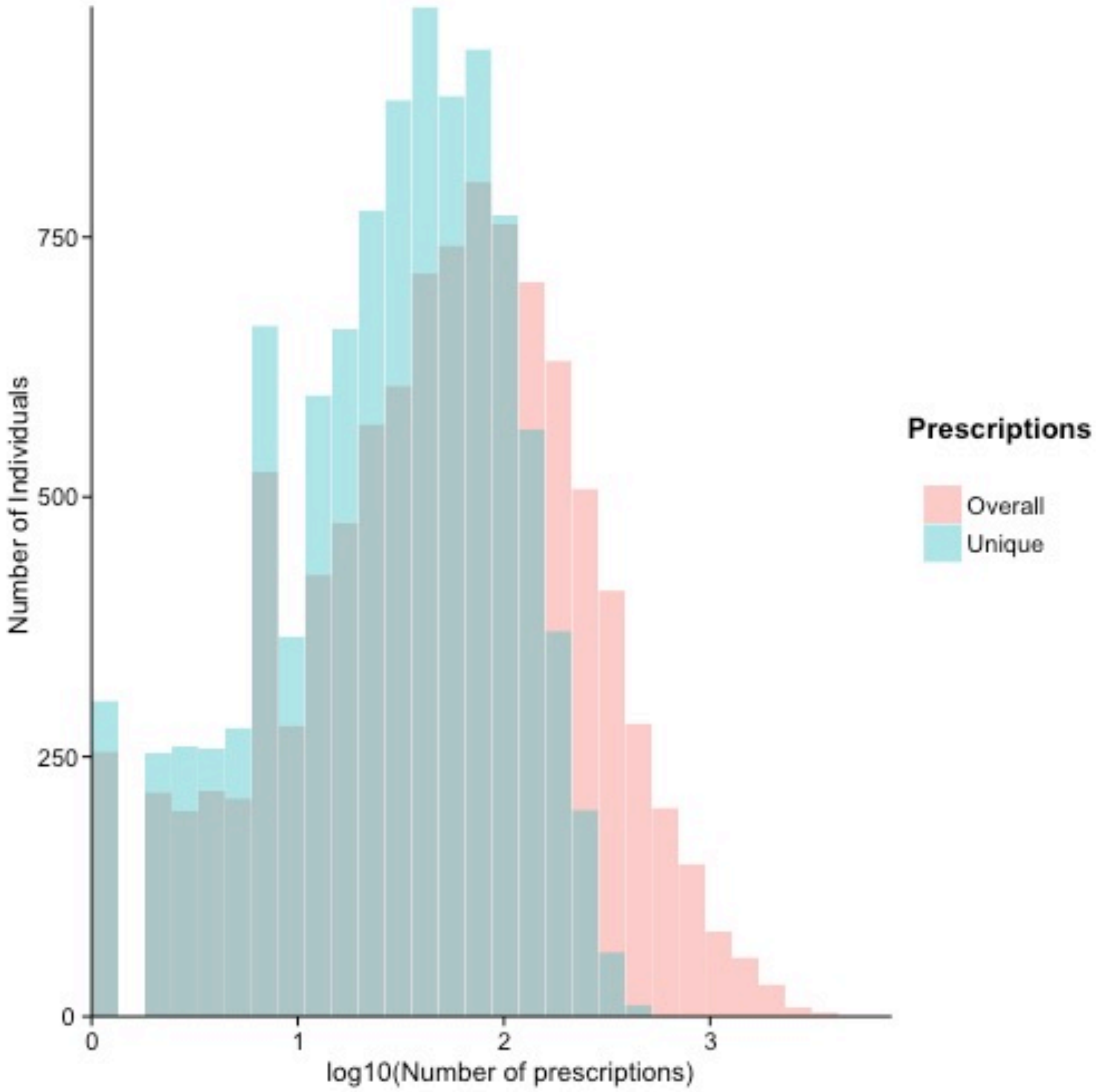

Supplementary Figure 4.

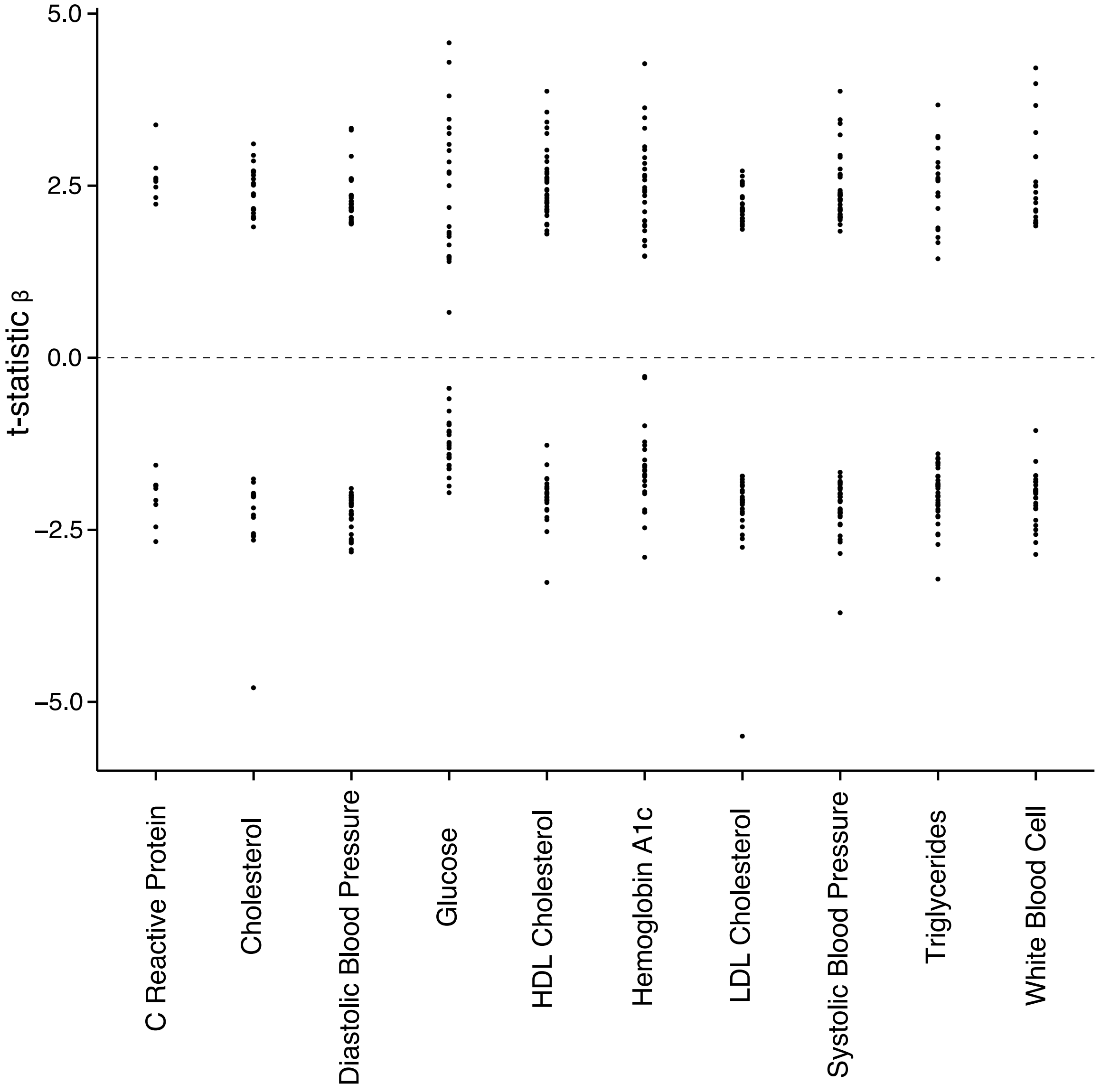

Supplementary Figure 5.

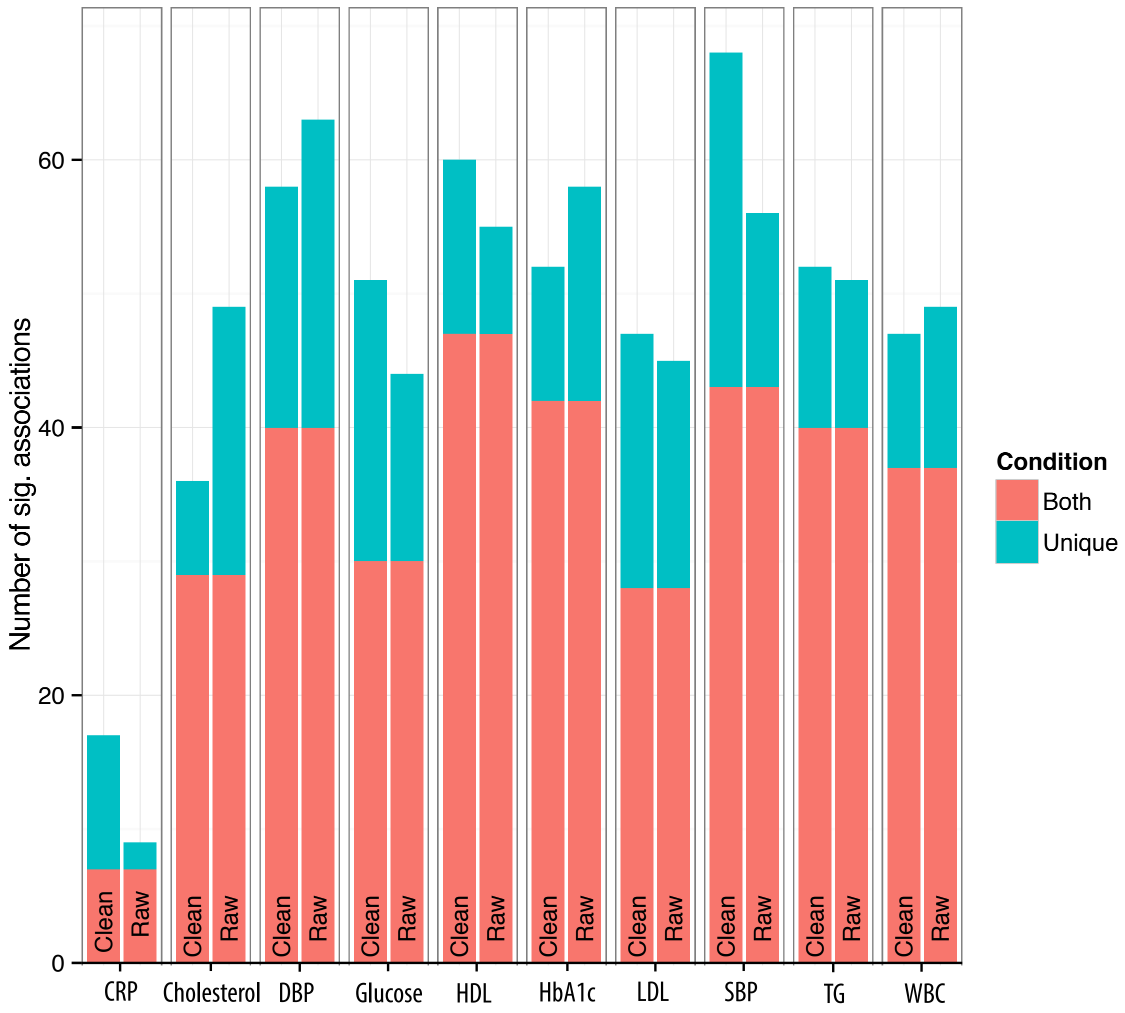

Supplementary Figure 6.

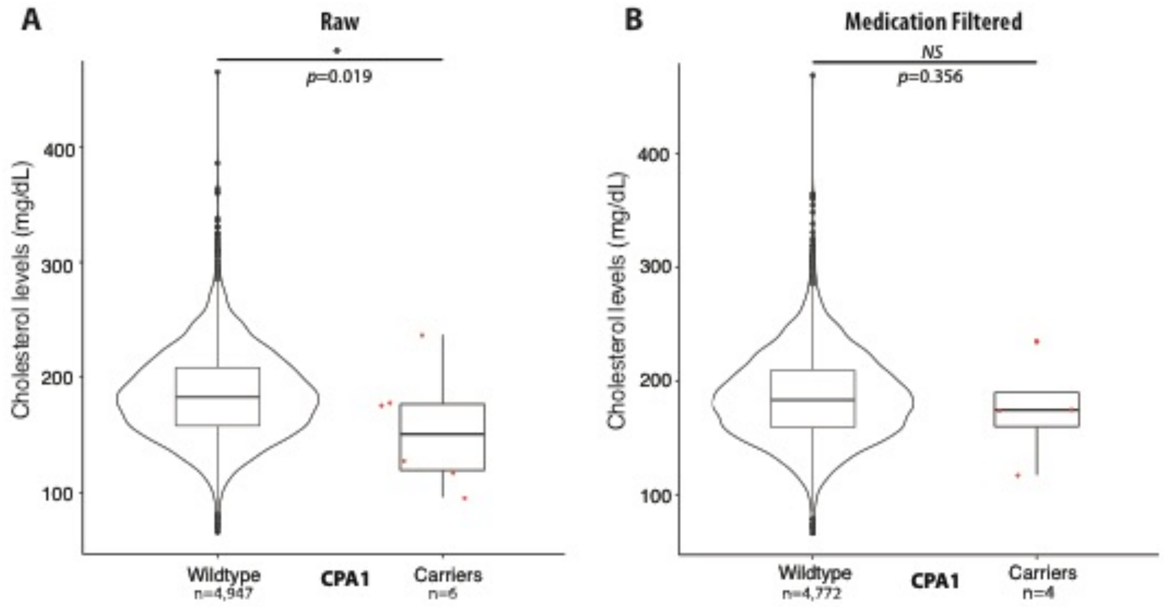

Supplementary Figure 7.

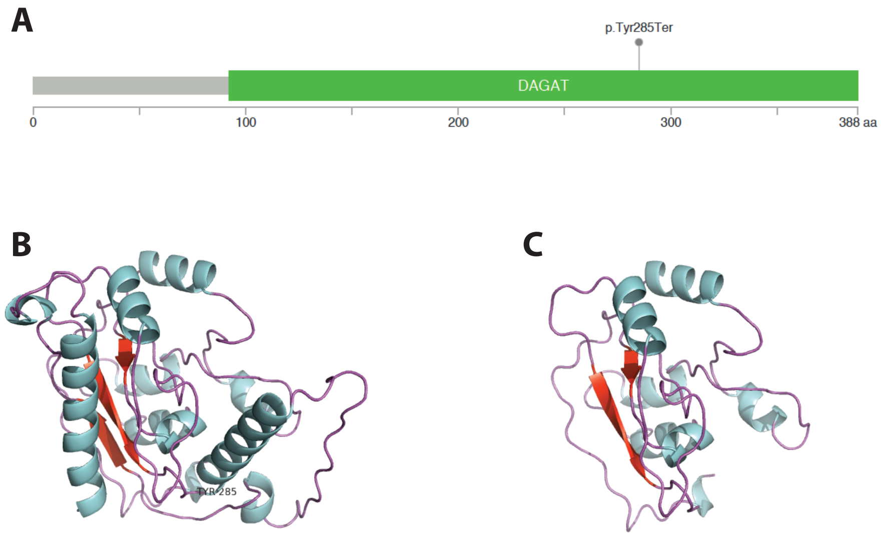

Supplementary Figure 8 (A).

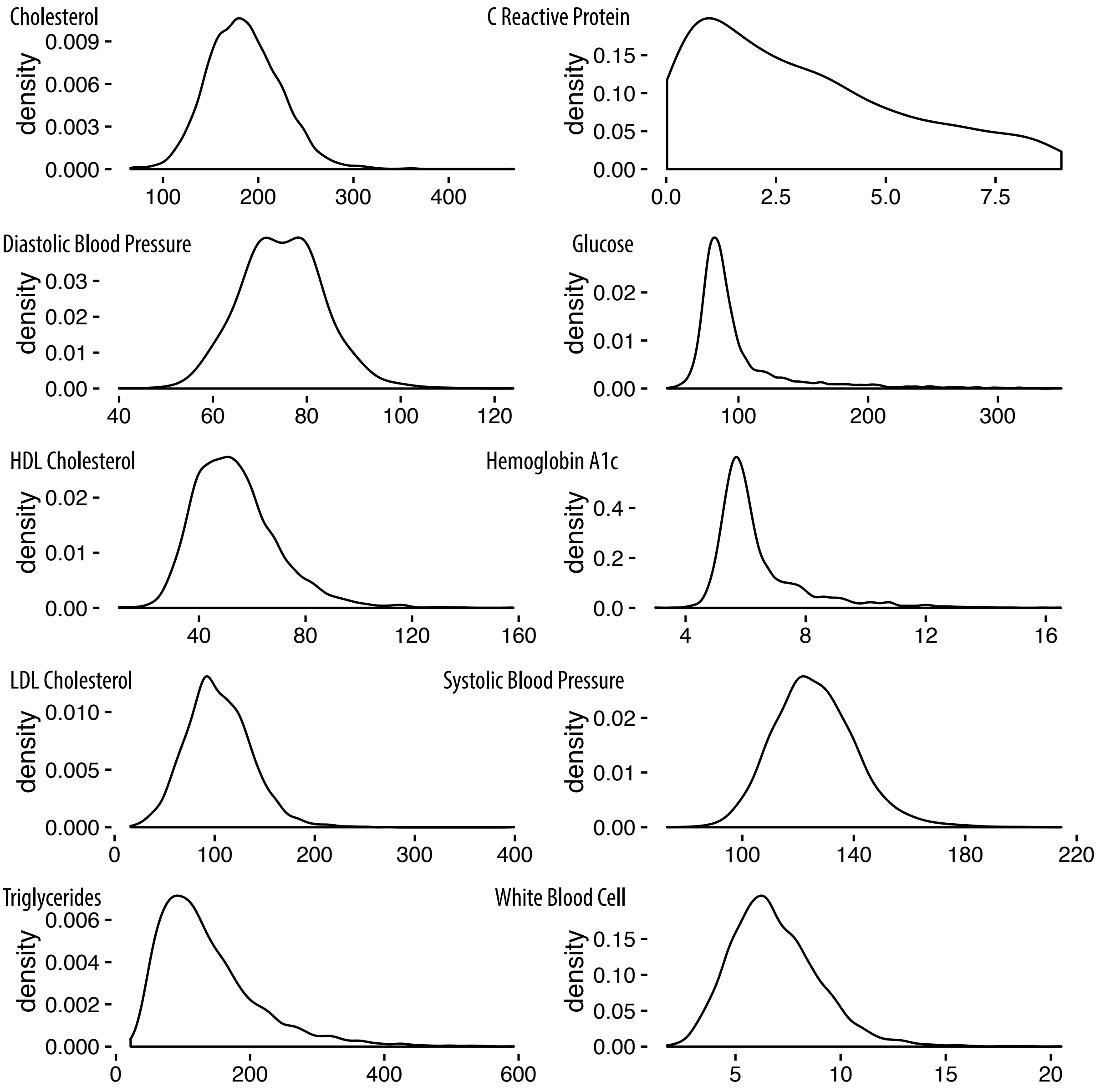

Supplementary Figure 8 (B).

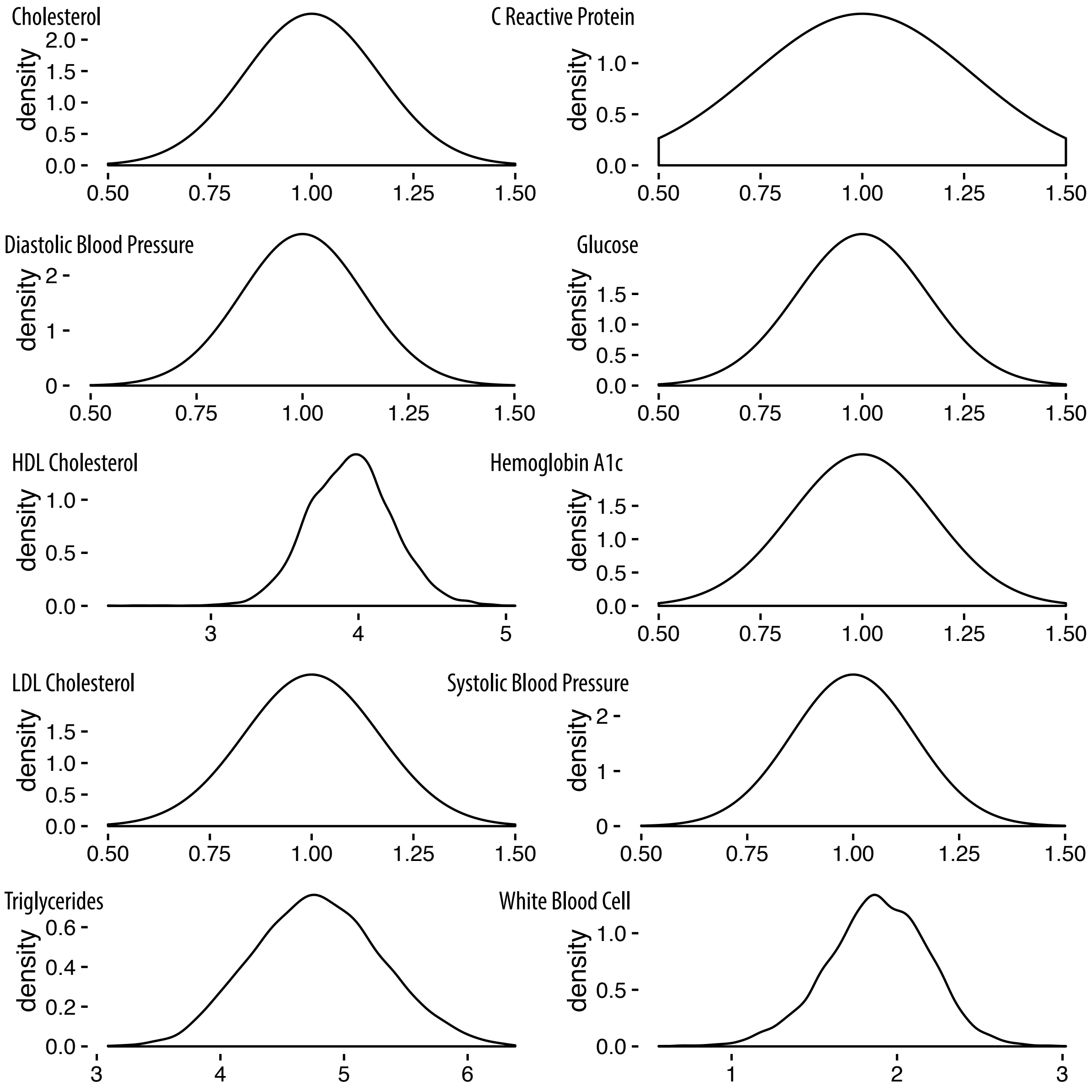

Supplementary Figure 8 (C).

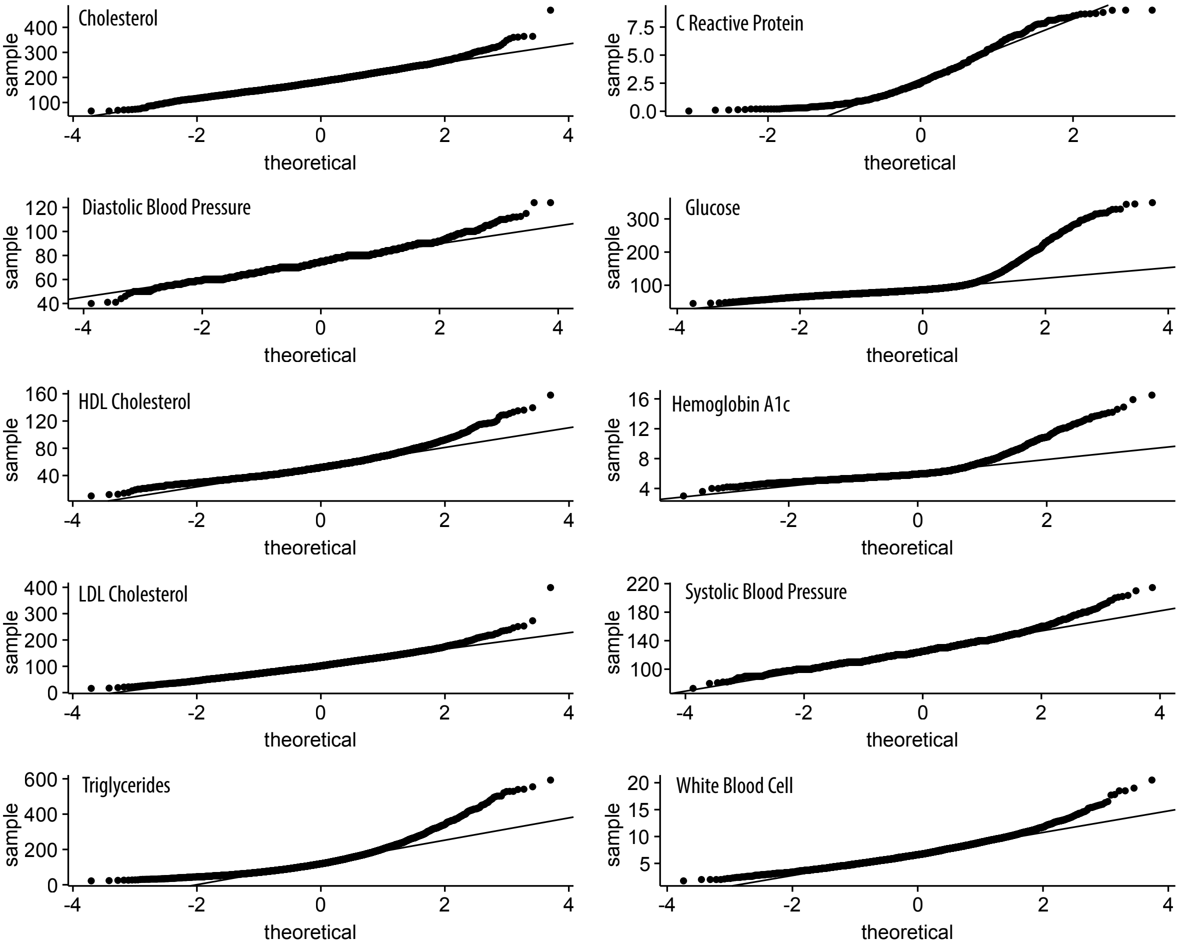

Supplementary Figure 8 (D).

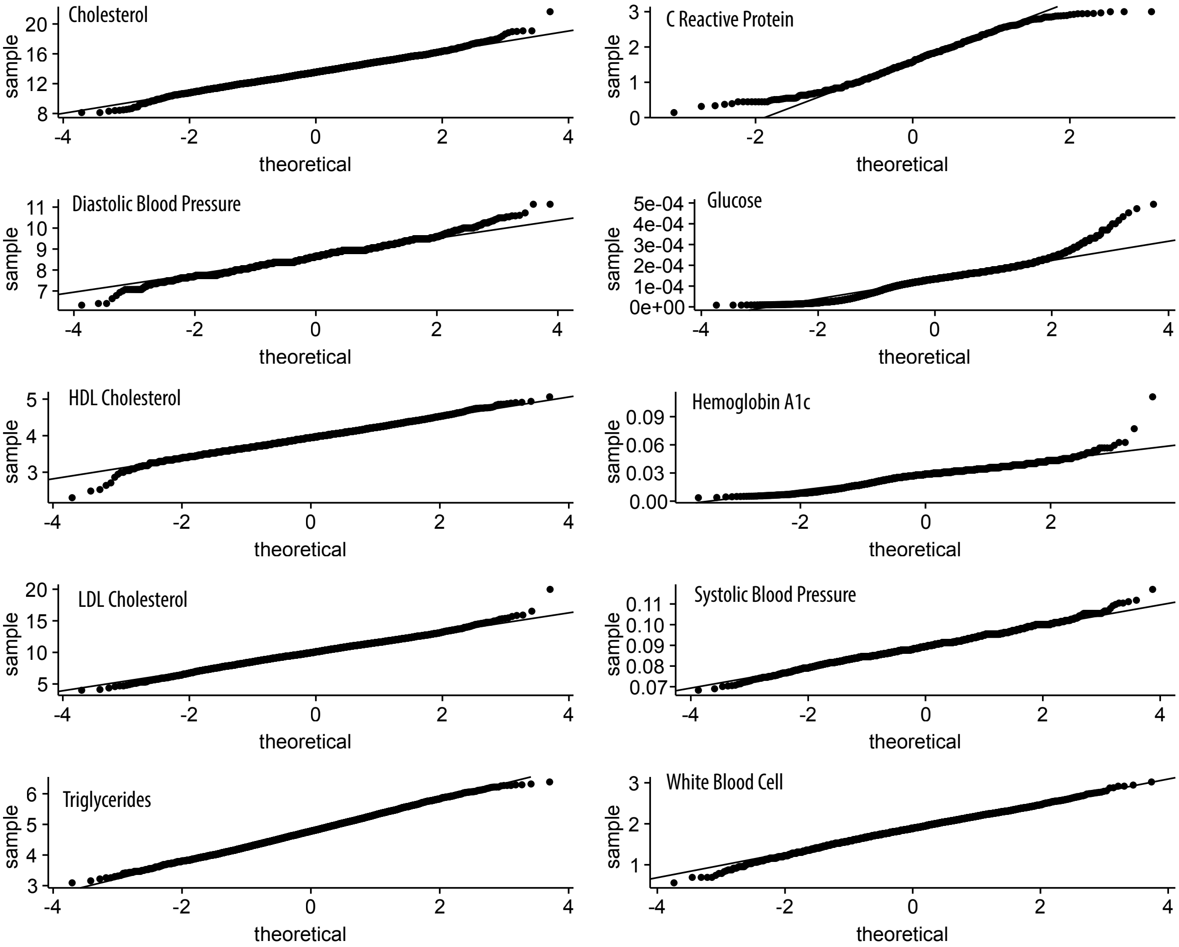

Supplementary Figure 9

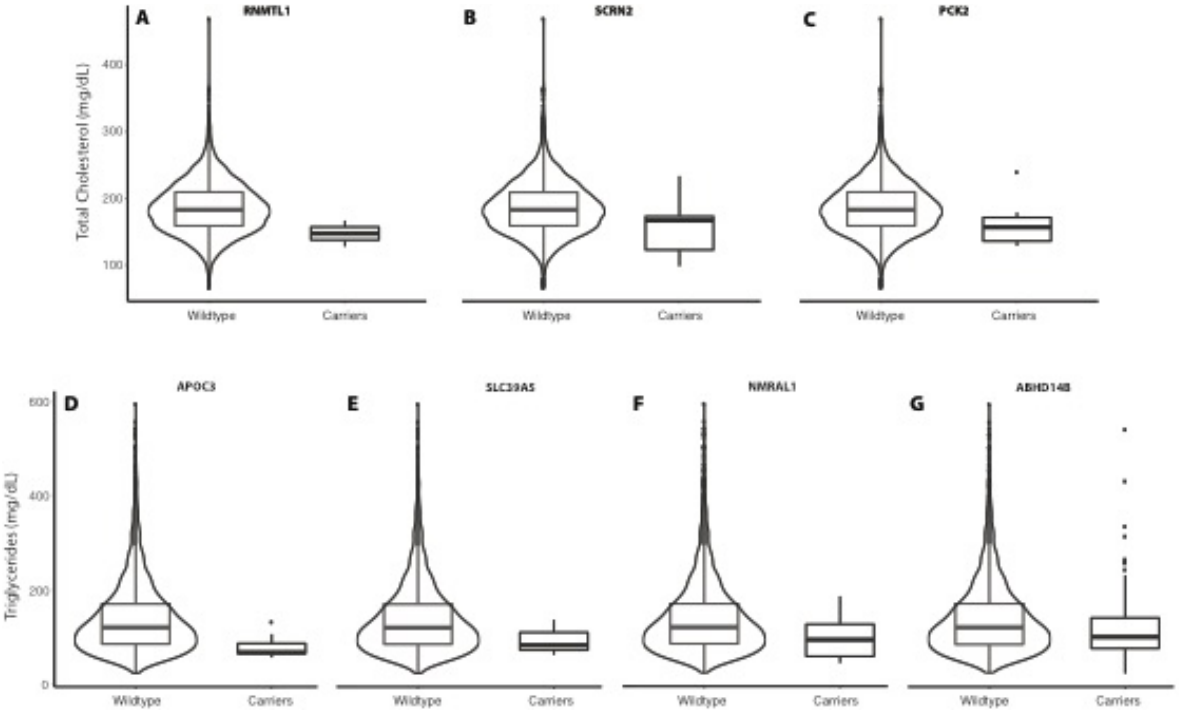

SUPPLEMENTARY REFERENCES

1. Wang GT, Peng B, Leal SM. Variant association tools for quality control and analysis of large-scale sequence and genotyping array data. American journal of human genetics. 2014;94(5):770-83. doi:10.1016/j.ajhg.2014.04.004.
2. Wang K, Li M, Hakonarson H. ANNOVAR: functional annotation of genetic variants from high-throughput sequencing data. Nucleic acids research. 2010;38(16):e164. doi:10.1093/nar/gkq603.
3. Cingolani P, Platts A, Wang le L, Coon M, Nguyen T, Wang L et al. A program for annotating and predicting the effects of single nucleotide polymorphisms, SnpEff: SNPs in the genome of Drosophila melanogaster strain w1118; iso-2; iso-3. Fly. 2012;6(2):80-92. doi:10.4161/fly.19695.
4. Patterson N, Price AL, Reich D. Population structure and eigenanalysis. PLoS genetics. 2006;2(12):e190. doi:10.1371/journal.pgen.0020190.
5. Abecasis GR, Auton A, Brooks LD, DePristo MA, Durbin RM, Handsaker RE et al. An integrated map of genetic variation from 1,092 human genomes. Nature. 2012;491(7422):56-65. doi:10.1038/nature11632.
6. Warren HR, Evangelou E, Cabrera CP, Gao H, Ren M, Mifsud B et al. Genome-wide association analysis identifies novel blood pressure loci and offers biological insights into cardiovascular risk. Nature genetics. 2017;49(3):403-15. doi:10.1038/ng.3768.
7. Gene Ontology Consortium: going forward. Nucleic acids research. 2015;43(Database issue):D1049-56. doi:10.1093/nar/gku1179.
8. Lee KW, Okot-Kotber C, LaComb JF, Bogenhagen DF. Mitochondrial ribosomal RNA (rRNA) methyltransferase family members are positioned to modify nascent rRNA in foci near the mitochondrial DNA nucleoid. The Journal of biological chemistry. 2013;288(43):31386-99. doi:10.1074/jbc.M113.515692.
9. Rorbach J, Boesch P, Gammage PA, Nicholls TJ, Pearce SF, Patel D et al. MRM2 and MRM3 are involved in biogenesis of the large subunit of the mitochondrial ribosome. Mol Biol Cell. 2014;25(17):2542-55. doi:10.1091/mbc.E14-01-0014.
10. Xu J, De Zhu J, Ni M, Wan F, Gu JR. The ATF/CREB site is the key element for transcription of the human RNA methyltransferase like 1(RNMTL1) gene, a newly discovered 17p13.3 gene. Cell research. 2002;12(3-4):177-97. doi:10.1038/sj.cr.7290124.
11. Way G, Morrice N, Smythe C, O'Sullivan AJ. Purification and identification of secernin, a novel cytosolic protein that regulates exocytosis in mast cells. Molecular biology of the cell. 2002;13(9):3344-54. doi:10.1091/mbc.E01-10-0094.
12. Stewart TP, Kim HY, Saxton AM, Kim JH. Genetic and genomic analysis of hyperlipidemia, obesity and diabetes using (C57BL/6J × TALLYHO/JngJ) F2 mice. BMC genomics. 2010;11:713. doi:10.1186/1471-2164-11-713.
13. Modaressi S, Brechtel K, Christ B, Jungermann K. Human mitochondrial phosphoenolpyruvate carboxykinase 2 gene. Structure, chromosomal localization and tissue-specific expression. The Biochemical journal. 1998;333 (Pt 2):359-66.
14. Geiser J, De Lisle RC, Andrews GK. The zinc transporter Zip5 (Slc39a5) regulates intestinal zinc excretion and protects the pancreas against zinc toxicity. PloS one. 2013;8(11):e82149. doi:10.1371/journal.pone.0082149.
15. Wang F, Kim B-EE, Petris MJ, Eide DJ. The mammalian Zip5 protein is a zinc transporter that localizes to the basolateral surface of polarized cells. The Journal of biological chemistry. 2004;279(49):51433-41. doi:10.1074/jbc.M408361200.
16. Guo H, Jin X, Zhu T, Wang T, Tong P, Tian L et al. SLC39A5 mutations interfering with the BMP/TGF-beta pathway in non-syndromic high myopia. Journal of medical genetics. 2014;51(8):518-25. doi:10.1136/jmedgenet-2014-102351.

17. Padmanabhan B, Kuzuhara T, Adachi N, Horikoshi M. The crystal structure of CCG1/TAF(II)250-interacting factor B (CIB). The Journal of biological chemistry. 2004;279(10):9615-24. doi:10.1074/jbc.M312165200.
18. Posorski N, Kaemmerer D, Ernst G, Grabowski P, Hoersch D, Hommann M et al. Localization of sporadic neuroendocrine tumors by gene expression analysis of their metastases. Clinical & experimental metastasis. 2011;28(7):637-47. doi:10.1007/s10585-011-9397-5.
19. Marin-Buera L, Garcia-Bartolome A, Moran M, Lopez-Bernardo E, Cadenas S, Hidalgo B et al. Differential proteomic profiling unveils new molecular mechanisms associated with mitochondrial complex III deficiency. Journal of proteomics. 2015;113:38-56. doi:10.1016/j.jprot.2014.09.007.
20. Zhao Y, Zhang J, Li H, Li Y, Ren J, Luo M et al. An NADPH sensor protein (HSCARG) down-regulates nitric oxide synthesis by association with argininosuccinate synthetase and is essential for epithelial cell viability. The Journal of biological chemistry. 2008;283(16):11004-13. doi:10.1074/jbc.M708697200.
21. Gan Q, Li T, Hu B, Lian M, Zheng X. HSCARG inhibits activation of NF-kappaB by interacting with IkappaB kinase-beta. Journal of cell science. 2009;122(Pt 22):4081-8. doi:10.1242/jcs.054007.
22. Lian M, Zheng X. HSCARG regulates NF-kappaB activation by promoting the ubiquitination of RelA or COMMD1. The Journal of biological chemistry. 2009;284(27):17998-8006. doi:10.1074/jbc.M809752200.
23. 1000 Genomes Project Consortium, Auton A, Brooks LD, Durbin RM, Garrison EP, Kang HM et al. A global reference for human genetic variation. Nature. 2015;526(7571):68-74. doi:10.1038/nature15393.
24. Lek M, Karczewski KJ, Minikel EV, Samocha KE, Banks E, Fennell T et al. Analysis of protein-coding genetic variation in 60,706 humans. Nature. 2016;536(7616):285-91. doi:10.1038/nature19057.
25. Exome Variant Server. NHLBI GO Exome Sequencing Project (ESP), Seattle, WA (URL: http://evs.gs.washington.edu/EVS/),.
